# Efficacy and safety of electroacupuncture-based comprehensive treatment for post-stroke depression: a systematic review and meta-analysis of randomized controlled trials

**DOI:** 10.3389/fpsyt.2025.1610032

**Published:** 2025-08-15

**Authors:** Xiaomei Hu, Yanying Pan, Yasi Tang, You Zhang, Zhaoping Liu, Yue Zhuo, Hong Zhang, Xiqin Yi

**Affiliations:** ^1^ College of Acupuncture, Massage, and Rehabilitation, Hunan University of Chinese Medicine, Changsha, Hunan, China; ^2^ College of Medical Imaging Laboratory and Rehabilitation, Xiangnan University, Chenzhou, Hunan, China

**Keywords:** post stroke depression, electroacupuncture, meta-analysis, randomized controlled trial, acupoint application

## Abstract

**Objective:**

This research aims to assess the therapeutic effects and safety of treatments for PSD by conducting a thorough systematic review and meta-analysis.

**Methods:**

Randomized controlled trials (RCTs) were retrieved from PubMed, Embase, Web of Science, The Cochrane Library, China National Knowledge Infrastructure (CNKI), and Wan Fang, covering all available records up to September 30, 2024. RCTs evaluating on the efficacy and safety of electroacupuncture in individuals with PSD were included. The robustness of the findings and possible contributors to heterogeneity were examined via sensitivity and subgroup analyses. Statistical analyses were conducted utilizing STATA 15.0 and Review Manager 5.4.

**Results:**

This study included 65 RCTs with a total of 5,362 participants. The results showed that electroacupuncture exhibited significantly greater clinical effectiveness compared to the control group (RR = 1.16, 95% CI [1.11, 1.22], *I*² = 59%, *p* < 0.00001), effectively reducing HAMD scores (SMD = -0.56, 95% CI [-0.72, -0.40], *I*² = 87%, *p* < 0.00001), SDS scores (SMD = -0.56, 95% CI [-0.87, -0.24], *I*² = 90%, *p* = 0.006), and TCM-DS scores (SMD = -0.52, 95% CI [-0.78, -0.27], *I*² = 0%, *p* < 0.0001). The incidence of adverse reactions was lower in the electroacupuncture (EA) intervention group (RR = 0.54, 95% CI [0.35, 0.83], I² = 0%, p = 0.004).The most commonly used acupoints were primarily located along the Gallbladder, Bladder, and Du Meridian, with the five most frequently used acupoints being: Baihui (GV20, 41 times), Shenting (DU24, 28 times), Taichong (LV3, 28 times), Shenmen (HT7, 26 times), and Neiguan (PC6, 22 times).

**Conclusion:**

Electroacupuncture could serve as a safe and effective complementary therapy for PSD. It is recommended that multicenter, large-scale, and high-quality RCTs be conducted to further validate these findings.

**Systematic review registration:**

https://www.crd.york.ac.uk/PROSPERO/view/CRD42024618618, identifier CRD42024618618

## 1 Introduction

Stroke ranks among the leading causes of death and long-term disability globally, causing significant neurological damage and functional limitations that greatly affect patients’ quality of life and recovery. In addition to neurological impairments, post-stroke depression (PSD) is a prevalent and concerning neuropsychiatric issue following a stroke (1). PSD is characterized by persistent depressive symptoms, emotional disturbances, and cognitive impairment, with a global prevalence estimated to range from 27% to 30% (2). The pathogenesis of PSD is complex, involving factors such as gender, a history of mental illness, the type of stroke, damage to the frontal and temporal lobes, insufficient social support, and severe physical disability, among others (3, 4). PSD often leads to a chronic and recurrent course, significantly impairing patients’ quality of life, delaying functional recovery, and increasing the risk of stroke recurrence and mortality. Current mainstream treatments still rely primarily on antidepressant medications and psychological therapies; however their efficacy is limited, and they are often accompanied by notable side effects—such as sleep disturbances, gastrointestinal discomfort, and sexual dysfunction—and even carry a risk of suicide triggered by overdose (5). Consequently, an increasing number of patients are turning to complementary and alternative therapies for managing PSD.

Electroacupuncture (EA), a modern technique combining traditional acupuncture with the delivery of low-frequency electrical pulses to specific acupoints, regulates the body’s electrical activity and energy flow, thereby achieving therapeutic benefits (6). Clinical studies have demonstrated that EA stimulation at acupoints on both the head—such as Baihui (GV20) and Yintang (GV29)—and the limbs—such as Taichong (LR3) and Hegu (LI4)—can significantly alleviate clinical symptoms in patients with mild to moderate post-stroke depression (PSD) (7, 8). Although the precise mechanism of action of EA is not yet fully understood, studies suggest that it may help restore neurological function and potentially stabilize post-stroke mood and cognition by modulating the central nervous system (9), reducing inflammation (10), and improving blood circulation (11).

To date, two systematic reviews on EA for the treatment of PSD have been published, one in 2018 (12) and the other in 2021 (13). These studies have summarized the existing evidence but ultimately reached contradictory conclusions. In addition, several new randomized controlled trials (RCTs) on EA for PSD have been conducted in recent years (7, 14). Accordingly, we conducted a meta-analysis and systematic review to assess safety and efficacy of EA in management of PSD, aiming to offer evidence-based guidance for its clinical use.

## 2 Materials and methods

### 2.1 Protocol and registration

This study followed the guidelines of the Preferred Reporting Items for Systematic Reviews and Meta-Analyses (PRISMA), including its protocols and extension statements for meta-analysis. The meta-analysis was registered in the International Prospective Register of Systematic Reviews (PROSPERO) with the registration number CRD42024618618.

### 2.2 Search methods

A thorough literature search was conducted across multiple databases, including PubMed, Embase, Web of Science (WOS), The Cochrane Library, Wan Fang, and China National Knowledge Infrastructure (CNKI), to identify RCTs published up to September 20, 2024. The focus was on studies evaluating the use of electroacupuncture as a complementary therapy for post-stroke depression. No language or geographical restrictions were applied to the search. The search terms included: “Electroacupuncture” and “Stroke” OR “Cerebrovascular Accident” OR “Cerebral Stroke” OR “Cerebrovascular Apoplexy” OR “Brain Vascular Accident” OR “Apoplexy” OR “CVA” (Cerebrovascular Accident) OR “Acute Stroke” OR “Acute Cerebrovascular Accident” AND “Depression” OR “Depressive Symptoms” OR “Depressive Disorder” AND “Randomized Controlled Trial” OR “Randomized Trials”. To ensure a comprehensive review, additional relevant studies were identified through a manual examination of the reference lists of articles meeting the inclusion criteria. The full search strategies can be found in **Supplementary Table S1**.

### 2.3 Study selection

Following a comprehensive evaluation process, RCTs were selected based on an assessment of titles, abstracts, and full texts. The selection process was guided by specific inclusion and exclusion criteria, outlined as follows:

Inclusion Criteria:

- Participants: The studies included adults aged 18 or older who were clinically diagnosed with PSD, with no restrictions on gender or ethnicity.
- Intervention: The experimental group must have used EA or EA combined with other therapies, such as traditional Chinese medicine, antidepressant medication, or rehabilitation.
- Control: The control group must have received non-electroacupuncture interventions.
- Outcomes: The study was required to report minimum one of the following primary outcome measures: HAMDS, SDS, or TCM-DS. The secondary outcome measures included the overall efficacy rate and adverse reaction rate. The criteria for the overall efficacy rate are provided in **Supplementary Table S2**.

Exclusion Criteria:

- Studies that are not RCTs, such as retrospective studies, animal experiments, or review articles.
- Studies involving participants diagnosed with depression unrelated to stroke, or who did not show depressive symptoms after having a stroke.
- Interventions in which the experimental group received EA in combination with other therapies not specified in the inclusion criteria.
- Studies with inaccurate data or incomplete outcome measurements, where missing data could not be obtained from the authors.
- Duplicate publications.

### 2.4 Data extraction

Two reviewers (PYY and TYS) independently conducted the initial screening of all retrieved literature using EndNote 9.0 software to eliminate duplicate studies. Subsequently, data were collected from the eligible studies, which included the following details:

Publication Details: Title, first author, and publication year.

Study Characteristics: Study design and duration of treatment.

Participant Information: Sample size gender, age, disease duration,

Intervention and Control: The intervention group reveived EA treatment, including specific acupoints, intensity, and frequency; the control group underwent alternative treatments, with specifications on dosage and frequency of administration.

For continuous data, the mean and standard deviation were recorded, whereas for categorical data, the number of events and total sample size were extracted.

A second round of screening was performed to identify studies meeting the inclusion criteria through a review of titles and abstracts. For studies where eligibility was unclear, the full texts were reviewed. In cases of disagreement, the final decision was made by a third reviewer (ZY). Once the final set of studies was determined, relevant data were extracted and synthesized.

### 2.5 Quality assessment

The quality of the included studies and the data extraction process were assessed using the Cochrane Collaboration’s Risk of Bias Tool for RCTs. Two reviewers (ZY and ZH) assessed the quality of the selected studies separately, and their assessments were cross-checked. The evaluation covered seven domains: random sequence generation, allocation concealment, blinding of participants and personnel, completeness of outcome data, reporting bias, and other potential biases. The methodological quality of the studies was classified into three categories: “high risk of bias,” “low risk of bias,” and “unclear risk of bias.” If the two reviewers disagreed, the final decision was made by a third reviewer (LZP).

### 2.6 Statistical analysis

EndNote 9.0 was used for reference management, Excel for data organization, and RevMan 5.4 and Stata 15.0 for statistical analysis. For binary outcomes, the risk ratio (RR) was employed as the effect size statistic, while for continuous outcomes, the standardized mean difference (SMD) was calculated. Heterogeneity was assessed using Cochrane’s Q test and I² statistics, with a significance level of α = 0.05 for the meta-analysis. Sensitivity analysis was performed by excluding studies one at a time. Publication bias was assessed using funnel plots and Egger’s test. Furthermore, subgroup analyses were conducted to evaluate the stability of outcomes and identify potential sources of heterogeneity.

## 3.Results

### 3.1Literature search and selection results

**Figure 1:**
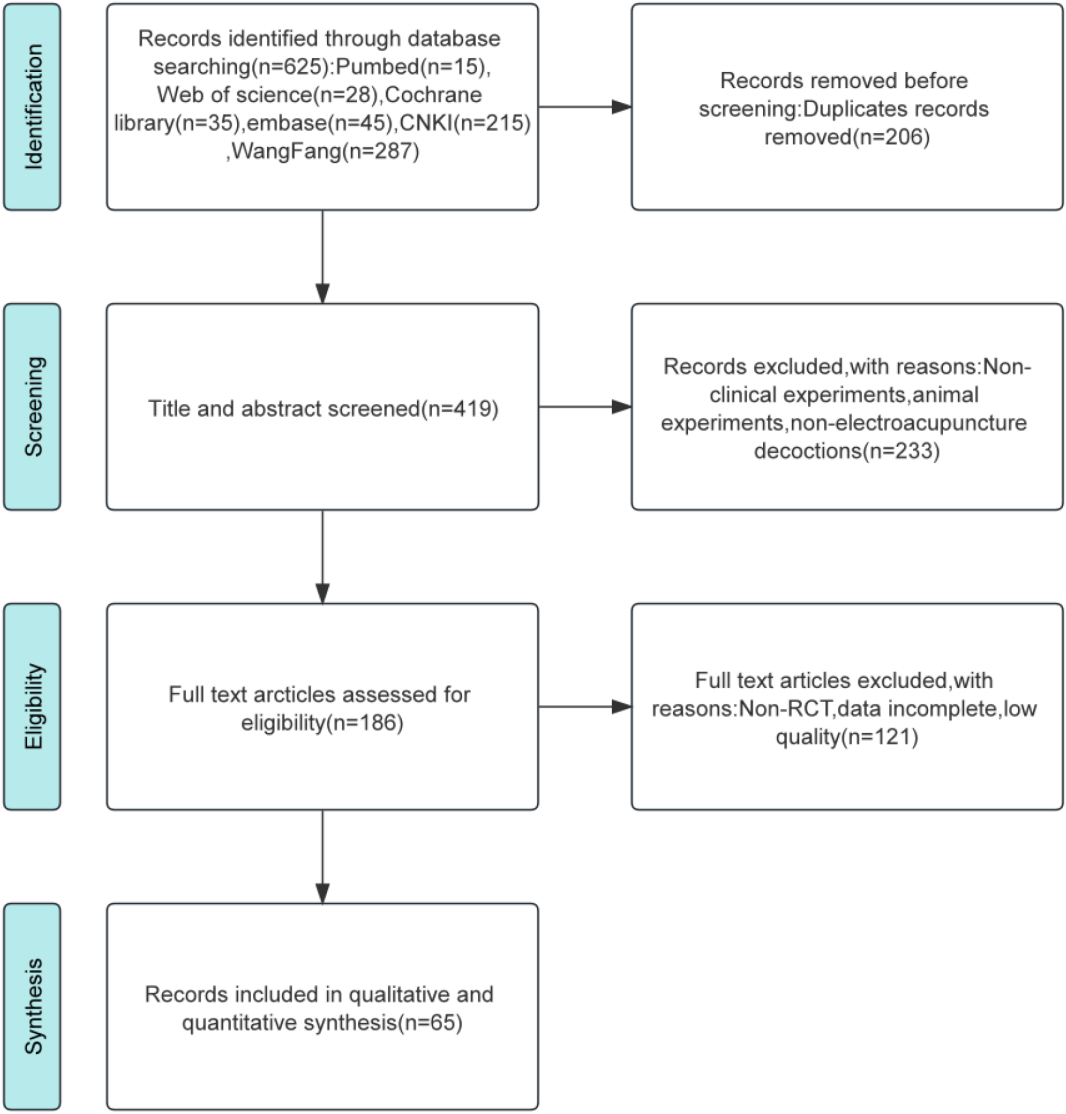
Flow chart of literature selection.

Through database searches, 625 relevant studies were initially identified. Following the removal of 206 duplicates, 419 studies were evaluated based on their titles and abstracts, leading to the exclusion of 233. The full-text review of the remaining 186 studies was conducted, resulting in the inclusion of 65 studies (14–78) (**Figure 1**).

### 3.2 Study characteristics

A total of 65 relevant studies were obtained through database retrieval, with 2,823 participants in the EA intervention group and 2,539 participants in the control group. Both the intervention and control groups each had sample sizes exceeding 40 participants. The average age of patients in the EA intervention group ranged from 42.38 to 72 years, and in the control group, it ranged from 43.17 to 73.7 years. The treatment duration varied between 4 and 12 weeks. The interventions in the EA intervention group included EA alone, EA combined with Chinese herbal medicine, EA combined with antidepressants, EA combined with conventional medicine, and EA combined with rehabilitation therapy, among others. The control group received treatments such as standard pharmacotherapy, rehabilitation, Chinese herbal medicine, or non-electroacupuncture needle therapy. Across the studies, a total of 57 acupuncture points were utilized for EA therapy, with the five most frequently used points being Bai Hui (GV 20), Shen Ting (GV 24), Tai Chong (LR 3), Shen Men (HT 7), and Nei Guan (PC 6). Details on study characteristics, criteria for clinical efficacy evaluation, intervention measures, outcomes, and acupuncture points are provided in **Supplementary Table S3**, **S4**. All acupuncture points referenced in the formulas adhered to the international standard nomenclature for acupuncture (79).

### 3.3 Bias risk assessment results

**Figure 2:**
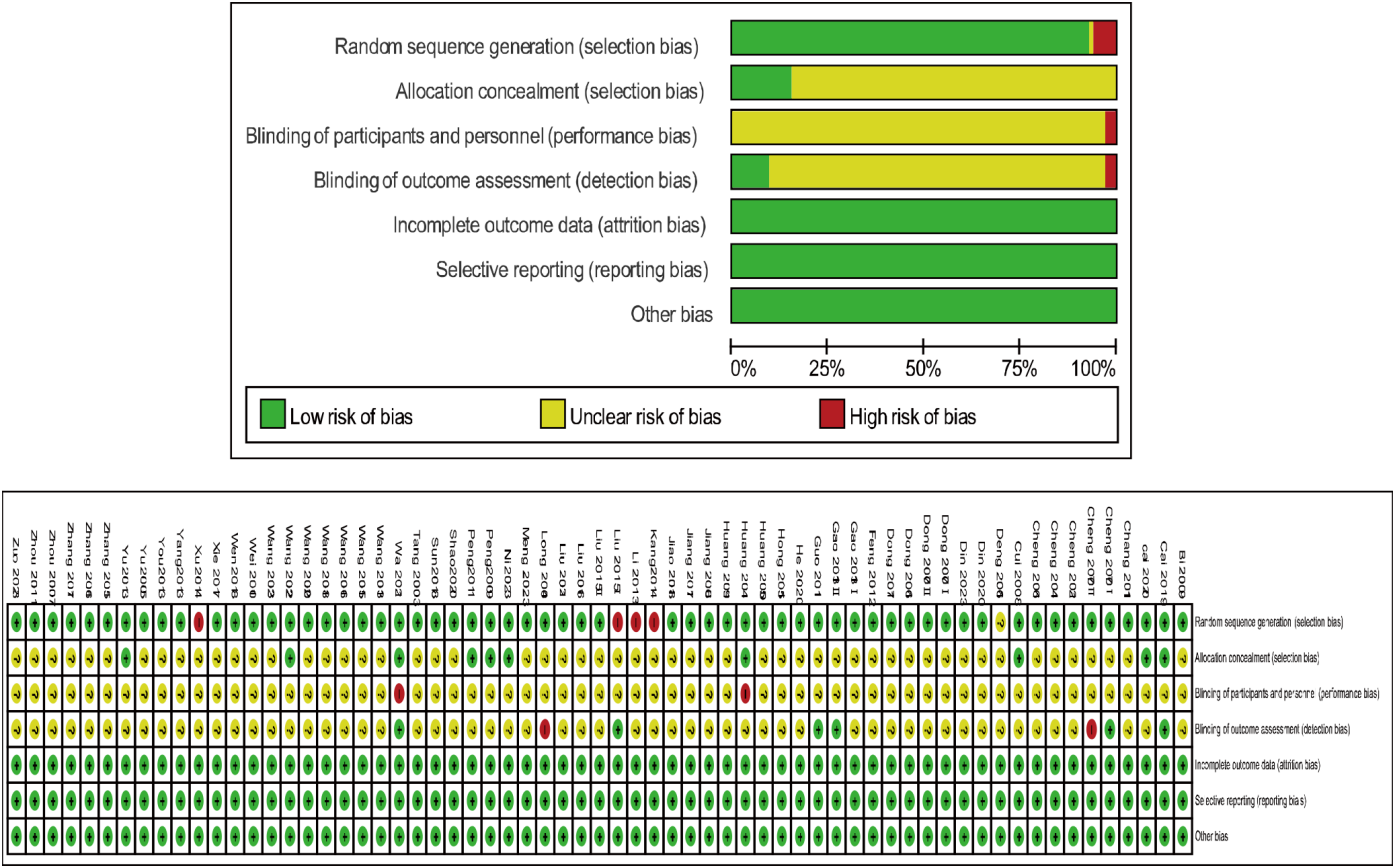
Bias risk assessment results.

The Cochrane Risk of Bias Assessment Tool was used to evaluate the quality of the included studies. Out of 65 studies, 60 employed random number tables for allocation, which were categorised as low risk of bias. One study (27) did not disclose the allocation method and was rated as unknown risk. Three studies (46, 47, 50) allocated participants by order of enrollment, and one study (67) allocated based on treatment, both rated as high risk of bias. Ten studies (14, 16–18, 41, 54–56, 70) explicitly reported allocation concealment and were categorized as low risk, while the remaining 55 studies were categorized as unknown risk. Two studies (18, 41) had non-blinding of participants and personnel, indicating high risk of bias, while 63 studies did not report blinding, rated as unknown risk. Additionally, five studies (17, 18, 24, 25, 46) reported blinding of outcomes and were rated low risk, while two studies (25, 52) had non-blinding of outcomes, rated high risk. Complete outcome data were available for all studies, which were rated as having a low risk of bias, with no other sources of bias identified (**Figure 2**).

### 3.4 Change in HAMD

**Figure 3:**
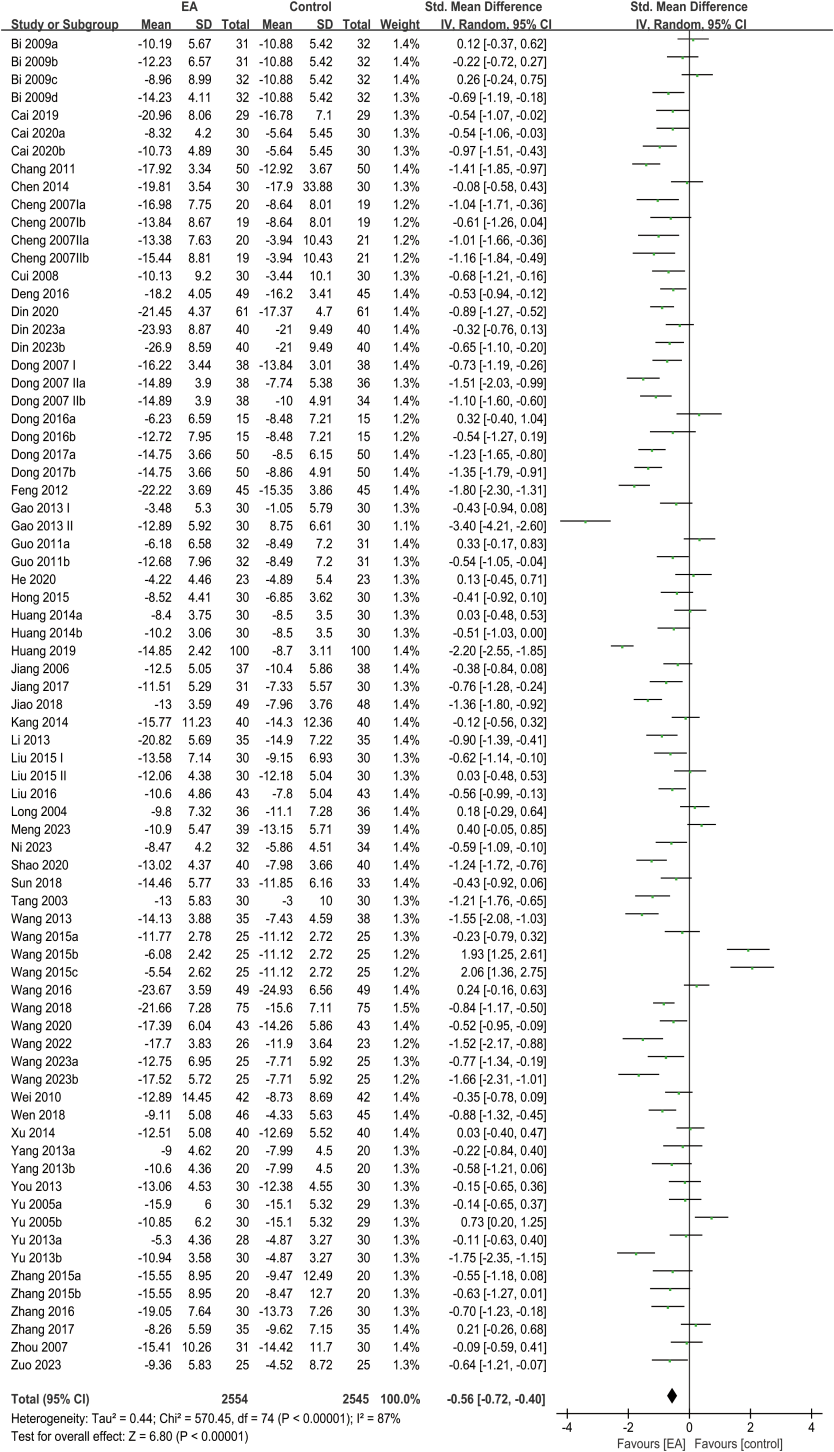
Forest plot of HAMD.

The analysis of clinical efficacy outcomes included a total of 75 studies. The findings showed that the EA intervention group exhibited a greater reduction in HAMD scores in individuals with PSD in comparison to the control group (SMD = -0.56, 95% CI [-0.72, -0.40], I² = 87%, p < 0.00001) (**Figure 3**).

Subgroup analyses were performed according to intervention types, treatment duration, and patient age. The results of the analysis revealed no significant difference in HAMD score reduction between the intervention and control groups in the EA combined with rehabilitation group, the EA combined with conventional medicine group, or the subgroup with an intervention duration greater than 6 weeks. In contrast, significant reductions in HAMD scores were observed in other subgroups, including the standalone EA group, EA combined with antidepressants, EA combined with both antidepressants and rehabilitation, EA combined with Chinese herbal medicine, and subgroups with intervention durations of 6 weeks or less, as well as those stratified by age (> 60 years and ≤ 60 years) (**Table 1**).

**Table 1:** HAMD subgroup analysis.

Subgroup	N	Heterogeneity analysis	Meta analysis	
		*I* ^2^(%)	SMD [95%CI]	*P* value
Total	75	87	-0.56 [-0.72, -0.40]	<0.00001
Intervention Method				
Electroacupuncture	19	84	-0.52 [-0.82, -0.22]	0.0007
Electroacupuncture + Rehabilitation	3	85	-0.41 [-1.04, 0.23]	0.21
Electroacupuncture + Pharmacotherapy	19	93	-0.56 [-0.99, -0.13]	0.01
Electroacupuncture + Pharmacotherapy + Rehabilitation	25	85	-0.58 [-0.85, -0.31]	<0.00001
Electroacupuncture + TCM	4	27	-0.42 [-0.75, -0.09]	0.01
Electroacupuncture + TCM + Pharmacotherapy + Rehabilitation	2	0	-0.58 [-0.89, -0.27]	0.0003
Intervention Time				
>6week	14	0	0.42 [0.16-1.12]	0.08
≤6week	61	88	-0.56 [-0.75, -0.37]	<0.00001
Mean/median age				
>60y	36	89	-0.43 [-0.69, -0.17]	0.001
≤60y	19	85	-0.92 [-1.21, -0.63]	<0.00001

### 3.5 Change in SDS

**Figure 4:**
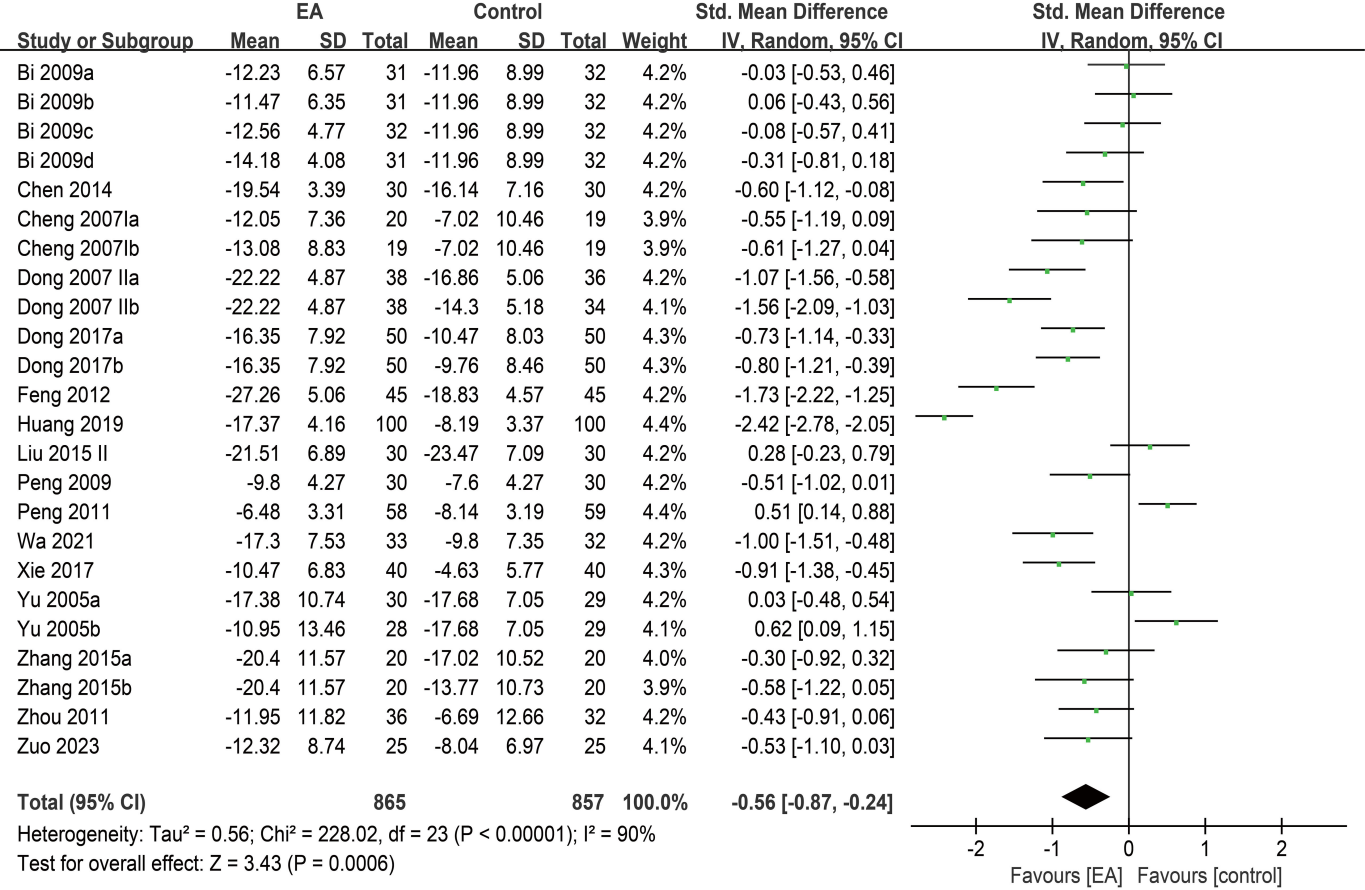
Forest plot of SDS.

The analysis of clinical efficacy outcomes included 24 studies in total. The results indicated that the EA intervention group achieved a significantly greater reduction in SDS scores in patients with PSD compared to the control group (SMD = -0.56, 95% CI [-0.87, -0.24], I² = 90%, p = 0.006) (**Figure 4**).

Subgroup analyses showed no statistically significant difference in SDS improvement between the intervention and control groups in the EA combined with rehabilitation group, the EA combined with antidepressants and rehabilitation group, the EA combined with Chinese herbal medicine group, the EA combined with conventional medicine group, and the subgroup with age > 60 years. In contrast, statistically significant reduction in SDS scores was observed in the remaining subgroups, including the standalone EA group, the EA combined with antidepressants group, the subgroup with intervention duration > 6 weeks, the subgroup with intervention duration ≤ 6 weeks, and the subgroup with age ≤ 60 years (**Table 2**).

**Table 2:** SDS subgroup analysis.

Subgroup	N	Heterogeneity analysis	Meta analysis	
		*I* ^2^(%)	SMD [95%CI]	*P* value
Total	24	90	-0.56 [-0.87, -0.24]	0.0006
Intervention Method				
Electroacupuncture	10	81	-0.53 [-0.90, -0.16]	0.005
Electroacupuncture + Rehabilitation	1	/	-0.08 [-0.57, 0.41]	0.74
Electroacupuncture + Pharmacotherapy	4	96	-1.28 [-2.37, -0.19]	0.02
Electroacupuncture + Pharmacotherapy+ Rehabilitation	6	81	-0.35 [-0.80, 0.10]	0.12
Electroacupuncture + TCM	3	57	-0.17 [-0.69, 0.35]	0.51
Intervention Time				
>6week	5	45	-0.31 [-0.64, 0.01]	0.06
≤6week	19	93	-0.61 [-0.99, -0.23]	0.002
Mean/median age				
>60y	10	95	-0.42 [-1.10, 0.26]	0.23
≤60y	6	76	-1.05 [-1.46, -0.64]	<0.00001

### 3.6 Change in TCM-DS

**Figure 5:**
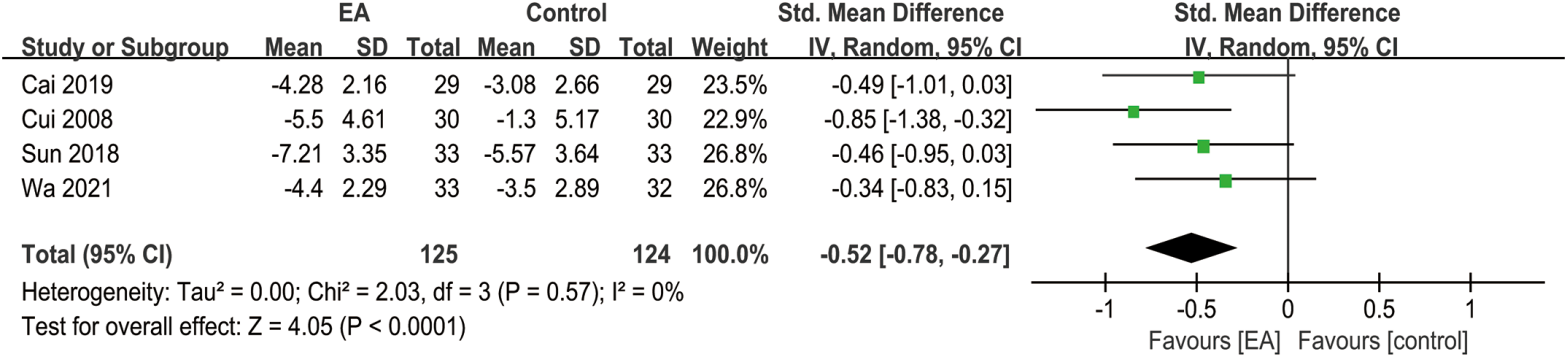
Forest plot of TCM-DS.

The analysis of clinical efficacy outcome measures included a total of 4 studies. The results indicated that the reduction in TCM-DS scores was more pronounced in the EA intervention group compared to the control group in patients with post-stroke depression (SMD = -0.52, 95% CI [-0.78, -0.27], *I*² = 0%, *p* < 0.0001) (**Figure 5**).

### 3.7 Overall efficacy rate

**Figure 6:**
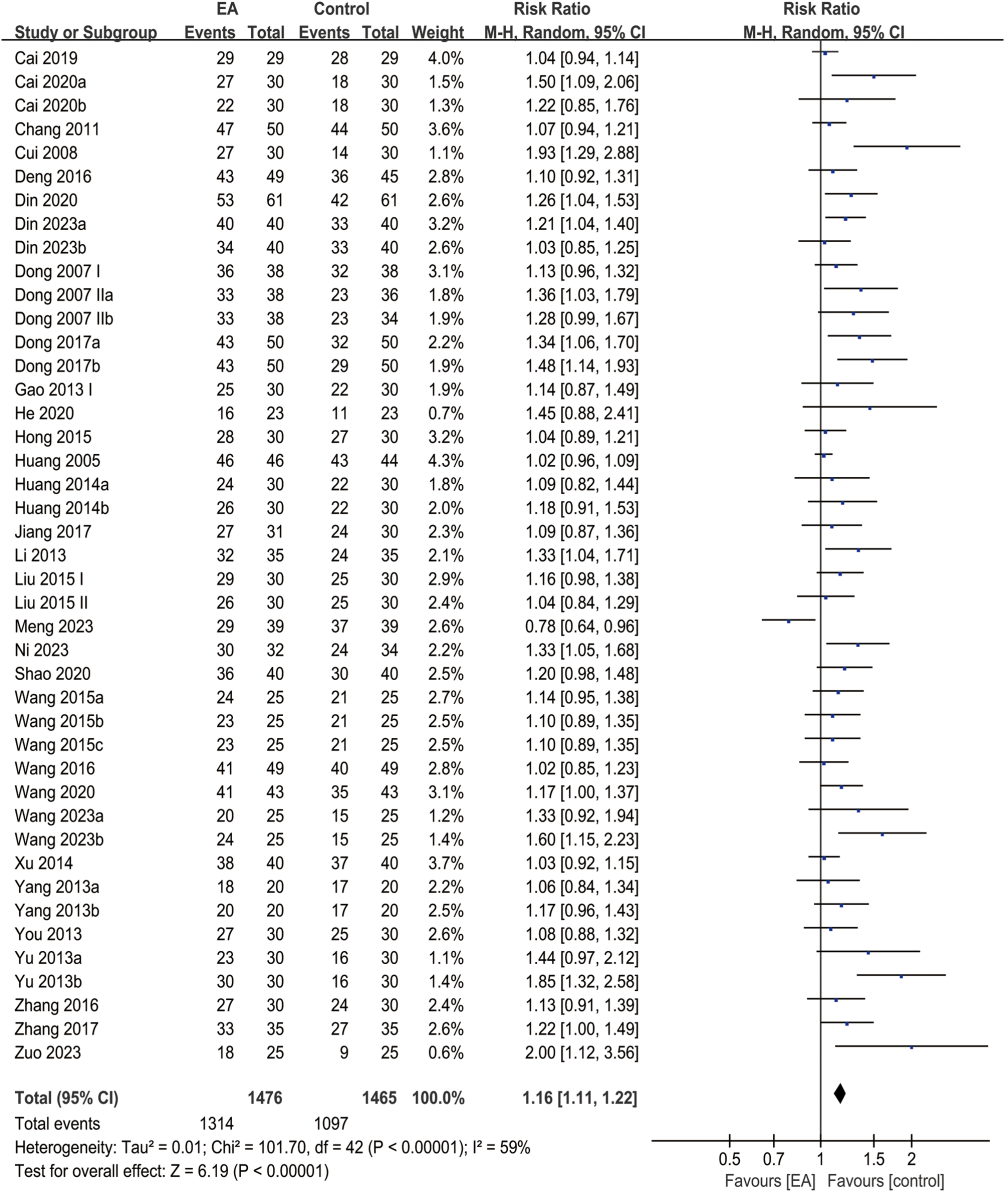
Forest plot of overall efficacy rate.

The analysis of clinical efficacy outcomes included 43 studies in total. The EA intervention group and control groups showed no significant difference in improving the overall efficacy rate for post-stroke depression. (RR = 1.16, 95% CI [1.11, 1.22], *I*² = 59%, *p* < 0.00001) (**Figure 6**).

### 3.8 Adverse reaction rate

**Figure 7:**
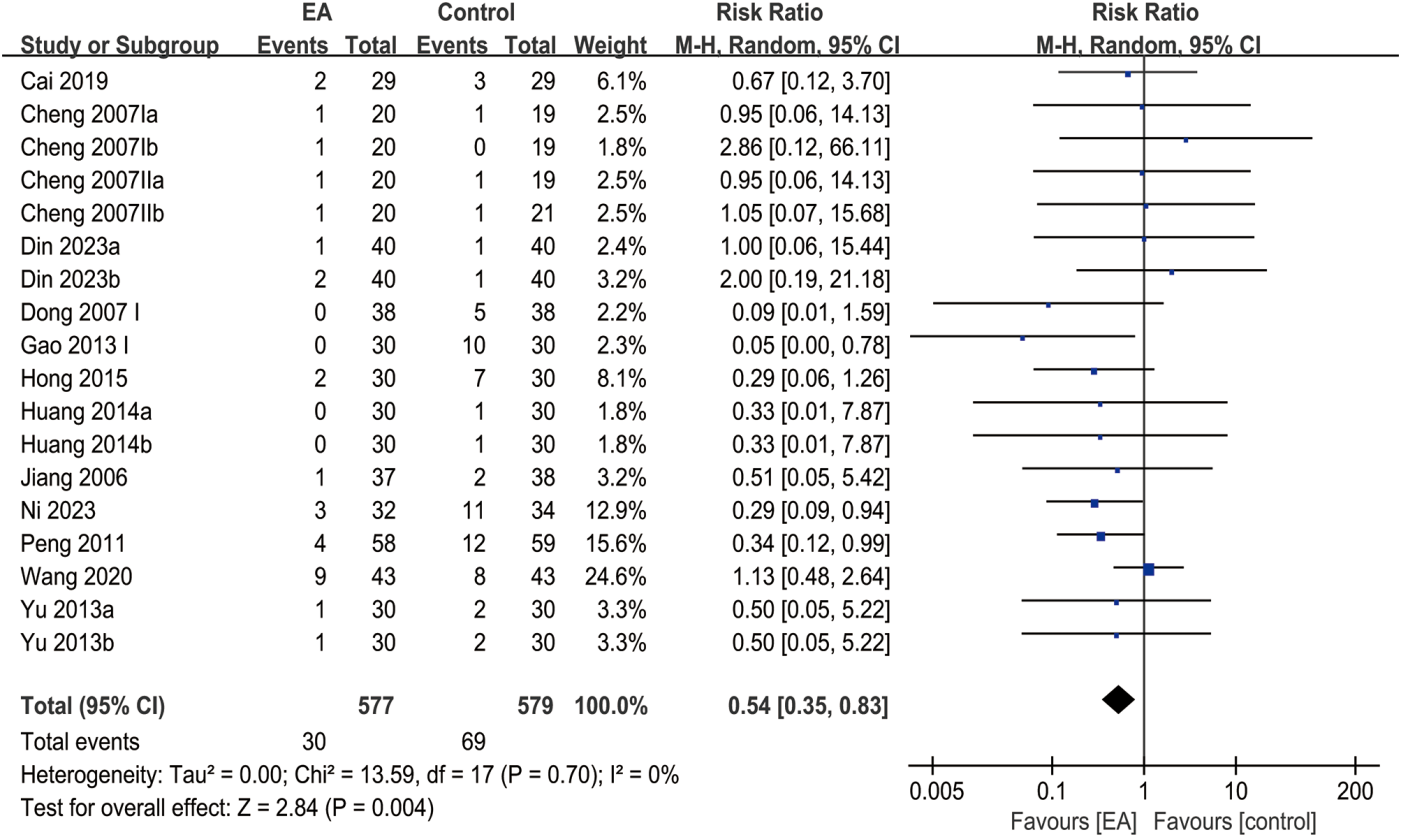
Forest plot of adverse reaction rate.

The analysis of clinical efficacy outcomes included a total of 18 studies. The results indicated that the adverse reaction rate of EA for post-stroke depression was significantly lower than that of the control group (RR = 0.54, 95% CI [0.35, 0.83], *I*² = 0%, *p* = 0.004) (**Figure 7**).

### 3.9 Publication bias and sensitivity analysis

**Figure 8:**
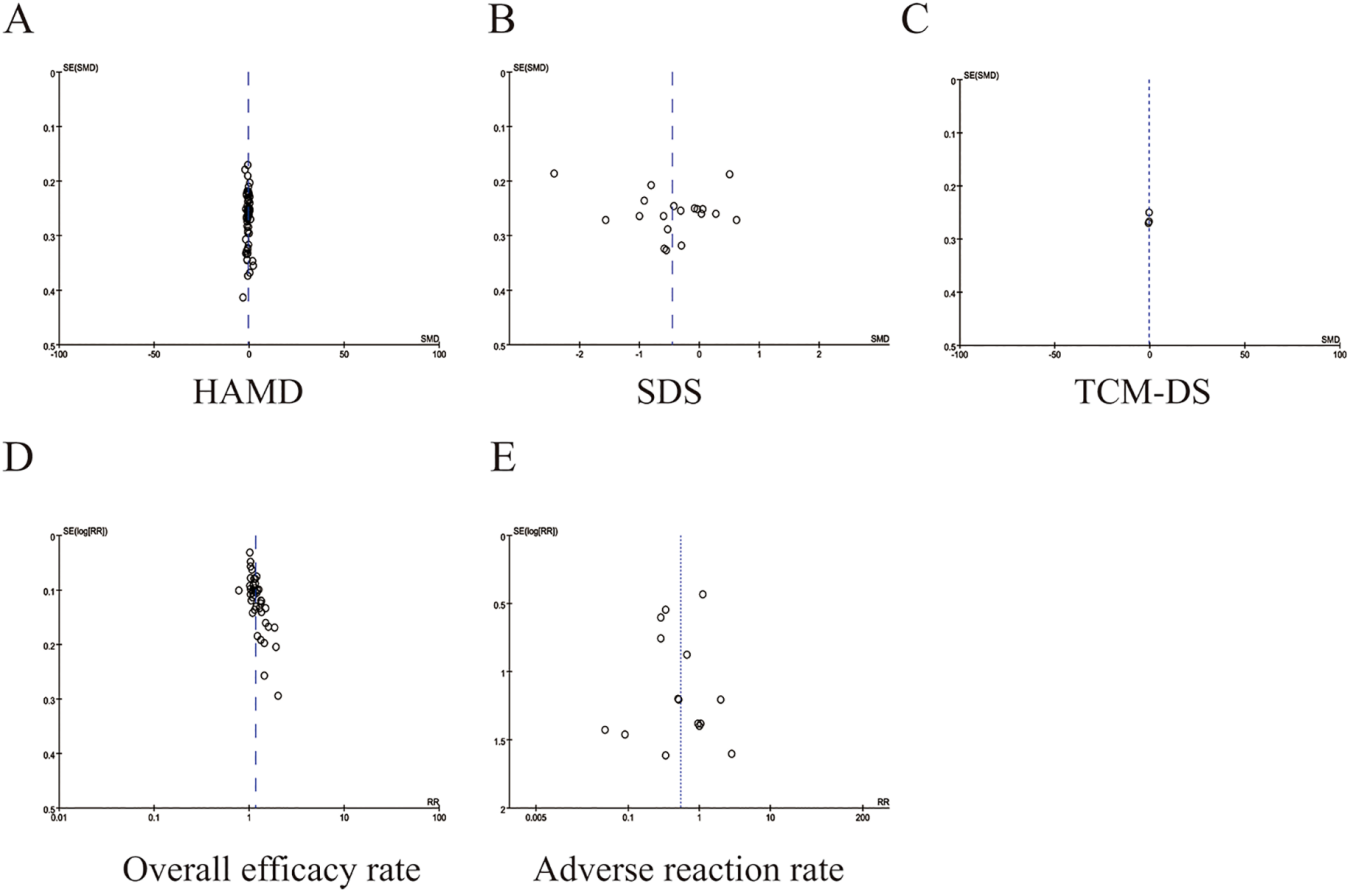
Funnel plots of outcomes. **(A)** HAMD **(B)** SDS **(C)** TCM-DS **(D)** Overall efficicacy rate **(E)** Aderverse reaction rate.

**Figure 9:**
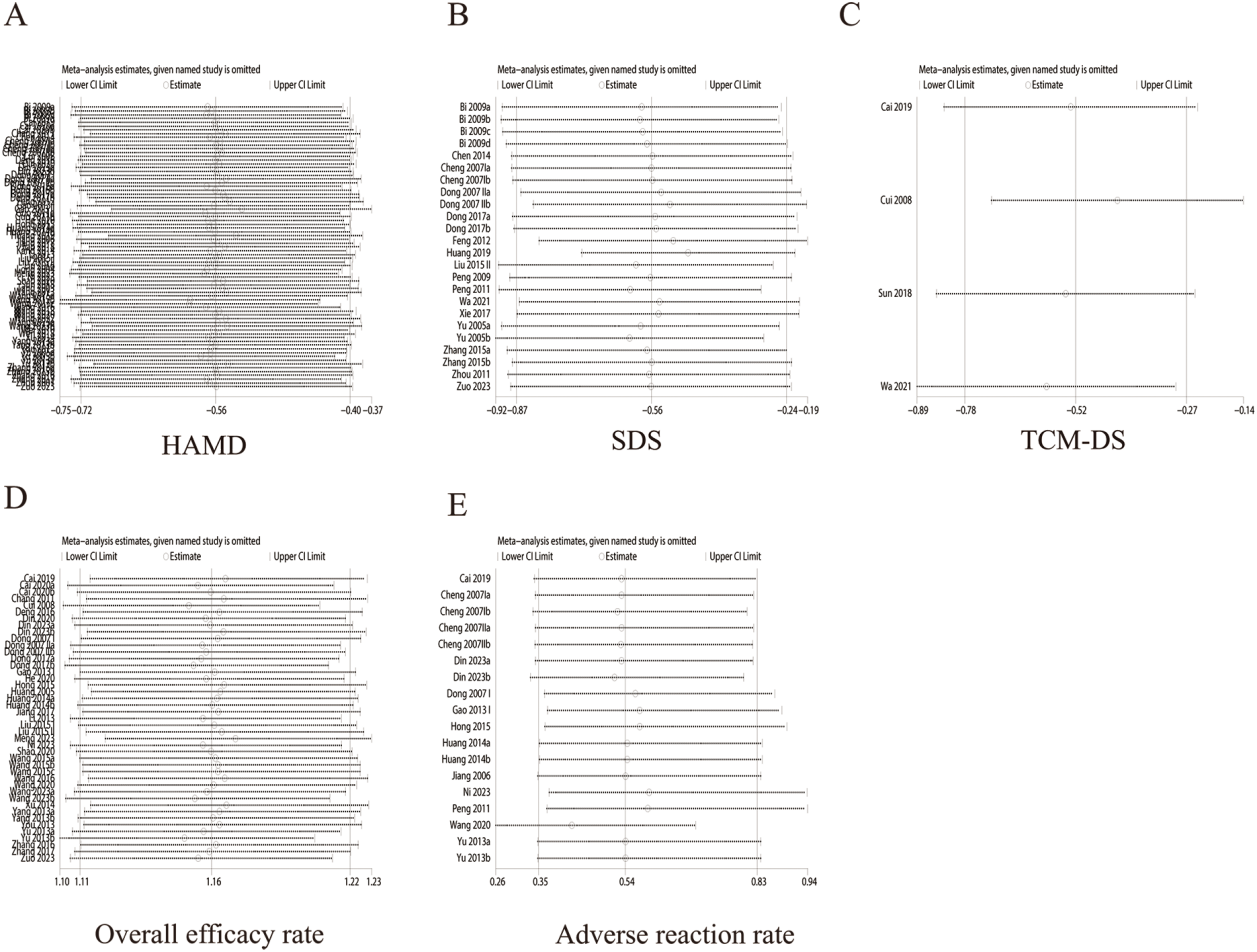
Sensitivity analysis of outcomes. **(A)** HAMD **(B)** SDS **(C)** TCM-DS **(D)** Overall efficicacy rate **(E)** Aderverse reaction rate.

The funnel plots for the overall efficacy rate displayed asymmetry (**Figure 8D**), suggesting potential publication bias. However, the funnel plots for other indicators, such as HAMD (**Figure 8A**), SDS (**Figure 8B**), TCM-DS (**Figure 8C**), and adverse reaction rate (**Figure 9E**), demonstrated acceptable symmetry. Egger’s test was used to evaluate publication bias, which revealed significant bias for the overall efficacy rate. (*p* = 0.0000), No bias was observed for HAMD, SDS, TCM-DS, or adverse reaction rate (*p* = 0.372, *p* = 0.334, *p* = 0.215, *p* = 0.163, *p* = 0.695). To assess the robustness of the results, sensitivity analyses were performed, and the results indicated that the sensitivity of all indicators, including HAMD (**Figure 9A**), SDS (**Figure 9B**), TCM-DS (**Figure 9C**), overall efficacy rate (**Figure 9D**), and adverse reaction rate (**Figure 9E**), remained stable. Sensitivity analysis details are provided in **Supplementary Table S5**.

## 4 Discussion

This meta-analysis provides several important findings. The EA intervention group demonstrated significant clinical improvements for patients with PSD, including reductions in HAMD, SDS and TCM-DS scores. Moreover, The EA intervention group showed a significantly lower incidence of adverse events compared to the control group. These results imply that EA may be an effective and safe treatment for alleviating depressive symptoms. Although the overall safety profile of EA was favorable, several trials reported mild and transient adverse effects, such as local redness, subcutaneous bruising, and vasovagal dizziness (17, 21, 22). These events were typically self-limiting and resolved with simple rest or local care. Therefore, ensuring proper aseptic technique, accurate point location, and standardized needle manipulation by trained practitioners is essential to minimize the occurrence of such adverse events (80).

To place our findings in the context of existing evidence, we reviewed two previously published meta-analyses on the efficacy of acupuncture for PSD.The first study (12) reported that no notable difference was identified between acupuncture and antidepressants in terms of improving depressive symptoms; the second study (13). indicated that acupuncture had effects on improving PSD that were comparable to those of antidepressants. Therefore, there is still no consensus regarding the efficacy of acupuncture in improving depressive symptoms. However, there are several notable differences between our study and these earlier analyses. First, the previous studies included 18 RCTs (n = 1,536 patients) and 19 RCTs (n = 1,606 patients), whereas our study incorporated 65 RCTs with a total of 5,362 participants. This substantial difference in sample size and study population could account for the differing results. Second, the earlier studies qualitatively assessed only one outcome (HAMD) and conducted subgroup analyses based solely on intervention duration. In contrast, our study evaluated quantitatively HAMD, SDS and TCM-DS outcomes and employed subgroup analyses based on intervention methods, intervention duration, and patient age, providing a more comprehensive understanding of EA’s efficacy. Third, we conducted sensitivity analyses and Egger’s tests to validate the robustness of our pooled results and assess the quality of evidence, which were not performed in the previous studies. Given these methodological advancements, the results of this analysis provide preliminary evidence supporting the efficacy of EA in the treatment of PSD; however, this evidence is limited by the overall quality of the included studies. High-quality randomized controlled trials are still needed to confirm these findings.

In clinical practice, both the HAMD and SDS scales are crucial tools for assessing the severity of depressive symptoms and the effectiveness of treatment in individuals with depression (81)Therefore, this study conducted subgroup analyses to comprehensively evaluate these two scales, identifying and exploring consistent trends in the changes observed in both the HAMD and SDS scores. Subgroup analysis, with intervention methods as a covariate, indicated the greatest improvements in both scales for the EA combined with antidepressants group, while the standalone EA group demonstrated the least improvement. This finding aligns with certain perspectives in complementary and alternative therapies for treating PSD (82, 83), reinforcing the potential benefits of EA in managing depression. Additionally, subgroup analysis based on intervention duration indicated that in the subgroup with intervention durations of ≤6 weeks, EA intervention group demonstrated consistent and significant improvements in both HAMD and SDS scores. Some clinical studies have found that EA for depression has a rapid onset, with effective symptom improvement occurring within 2 to 4 weeks (84, 85). However, at 8 weeks, although EA remains effective, some studies found no statistically significant differences (86). It is still difficult to determine to determine whether intervention durations of ≤ 6 weeks are indeed a key factor contributing to better outcomes, as there was a large discrepancy in sample sizes between the two subgroups. Therefore, further high-quality RCTs are neededto establish the optimal duration of EA treatment for PSD. Further analysis revealed that the duration of the intervention was a significant contributor to heterogeneity in this study. Finally, subgroup analysis by patient age indicated that the EA intervention group also had a notable therapeutic effect in the ≤ 60 years age group. Age is an important factor influencing the severity of depression and the effectiveness of acupuncture. Younger patients tend to have a lower incidence and severity of depression (87), Furthermore, some studies suggest that electroacupuncture may treat cerebrovascular diseases by affecting brain plasticity, while aging reduces brain plasticity, leading to a diminished therapeutic effect of electroacupuncture (88).

This study found that acupoints commonly used to alleviate PSD are primarily located along the Gallbladder, Bladder, and Du Meridians, and function to regulate Qi and blood, calm the mind, and relieve depression. The most frequently utilized acupoints included Bai hui (GV20, 41 times), Shen ting (DU24, 28 times), Tai chong (LR3, 28 times), Shen men (HT7, 26 times), and Nei guan (PC6, 22 times).

Bai hui (GV20)

GV20 is located on the Governing Vessel and is considered a critical convergence point of all meridians. Modern studies have demonstrated that EA stimulation of GV 20 can alleviate depressive symptoms by acutely activating the somatosensory and visual networks and, during the sustained phase, inhibiting the overactivation of the default mode network, suggesting both immediate and long-term therapeutic effects (89). Another study found that EA targeting GV20, LR3, Yintang (GV24), and Hegu (LI4) may reduce HAMD-17 scores by suppressing pro-inflammatory cytokines, with efficacy comparable to escitalopram oxalate (90).

Shenting (DU24)

DU 24, another acupoint on the Governing Vessel, is traditionally used to regulate emotions and is often combined with Baihui in the treatment of mood disorders. Studies have shown that EA stimulation of GV20 and DU24 alleviates depressive symptoms by modulating the expression of key proteins in the hippocampus, such as CaMKII, CaMKIV, and CaM, thereby reducing stress related to work and daily life (91). Electroacupuncture targeting GV20 and GV24 also alleviates depressive symptoms by activating the mesolimbic dopamine reward circuit, enhancing pain relief and emotional regulation (92).

Taichong (LR 3)

LR3 is located on the Liver meridian. Electroacupuncture at GV20 and LR3 alleviates depression through RNA sequencing technology, which identified changes in gene expression in the medial prefrontal cortex—including genes such as Casr, Bdkrb2, Gnb3, and Ccl1—suggesting that electroacupuncture modulates multiple biological pathways involved in the treatment of depression (93).

Shenmen (HT7)

HT7, located on the Heart meridian, is associated with calming the mind, benefiting the heart, and unblocking meridians. Research has shown that EA stimulation on HT7 alleviates anxiety and negative emotions induced by repeated alcohol administration by reducing plasma corticosterone levels, increasing amygdala expression of mature brain-derived neurotrophic factor and phosphorylated TrkB, and decreasing corticotropin-releasing hormone levels in the paraventricular nucleus, thereby modulating stress-related pathways (94).

Neiguan (PC6)

PC6, located on the Pericardium meridian, is known for its sedative and meridian-unblocking properties. Studies have shown that acupuncture stimulation of PC6 effectively alleviates depressive symptoms induced by chronic mild stress, including anxiety and anhedonia. This intervention restored behavioral indicators, such as open-arm latency in the elevated plus maze (EPM) test and sucrose intake, while significantly reducing the expression of c-fos in the paraventricular nucleus of the hypothalamus (95)

This study has certain limitations. Owing to the inherent characteristics of acupuncture interventions, most included trials lacked clear reporting of blinding and allocation procedures, as reflected in the quality assessment. Nevertheless, these methodological limitations did not substantially affect the overall findings. A large proportion of studies were sourced from Chinese databases, with small sample sizes and single-centre designs, potentially introducing publication and regional selection biases. Although acupuncture demonstrated a favourable safety profile across studies, it is noteworthy that only a small proportion of trials used sham acupuncture as a control, with the majority adopting active pharmacological comparators (**Supplementary Table S3**). This choice of control may have introduced selection bias and led to an overestimation of acupuncture’s relative safety. Further high-quality, rigorously designed trials are warranted to strengthen the evidence base.

## 5 Conclusion

Our analysis suggests that EA can alleviate PSD symptoms and enhance clinical efficacy, particularly when used in conjunction with antidepressants. However, the supporting evidence is limited due to the generally low quality of the RCTs considered, which reduces confidence in the observed benefits. EA also showed significant improvements in HAMD, SDS, and TCM-DS scores. Future research should prioritize high-quality, rigorously designed RCTs with robust controls, such as sham acupuncture, and longer follow-up periods to better evaluate long-term effects. Given the current evidence, the possible benefits of acupuncture for PSD should be approached with caution.

## Data Availability

The original contributions presented in the study are included in the article/**Supplementary Material**. Further inquiries can be directed to the corresponding authors.
