# Supplementary material for: Efficacy and safety of electroacupuncture-based comprehensive treatment for post-stroke depression: a systematic review and meta-analysis of randomized controlled trials
Source: Front Psychiatry. 2025 Aug 15;16:1610032. doi: 10.3389/fpsyt.2025.1610032 (PMC12395381; doi:10.3389/fpsyt.2025.1610032)

Table S1 Search strategy

Pubmed-15

****((("Electroacupuncture"[Mesh]) AND (("Stroke"[Mesh]) OR (((((((((((((((((Strokes) OR (Cerebrovascular Accident)) OR (Cerebrovascular Accidents)) OR (Cerebral Stroke)) OR (Cerebral Strokes)) OR (Cerebrovascular Apoplexy)) OR (Brain Vascular Accident)) OR (Brain Vascular Accidents)) OR (Cerebrovascular Stroke)) OR (Cerebrovascular Strokes)) OR (Apoplexy)) OR (CVA)) OR (CVAs)) OR (Acute Stroke)) OR (Acute Strokes)) OR (Acute Cerebrovascular Accident)) OR (Acute Cerebrovascular Accidents)))) AND (("Depression"[Mesh]) OR ((((Depressive Symptoms) OR (Depressive Symptom)) OR (Emotional Depression)) OR (Depressive Disorder)))) AND (random*)**** Sort by: ****Most Recent****
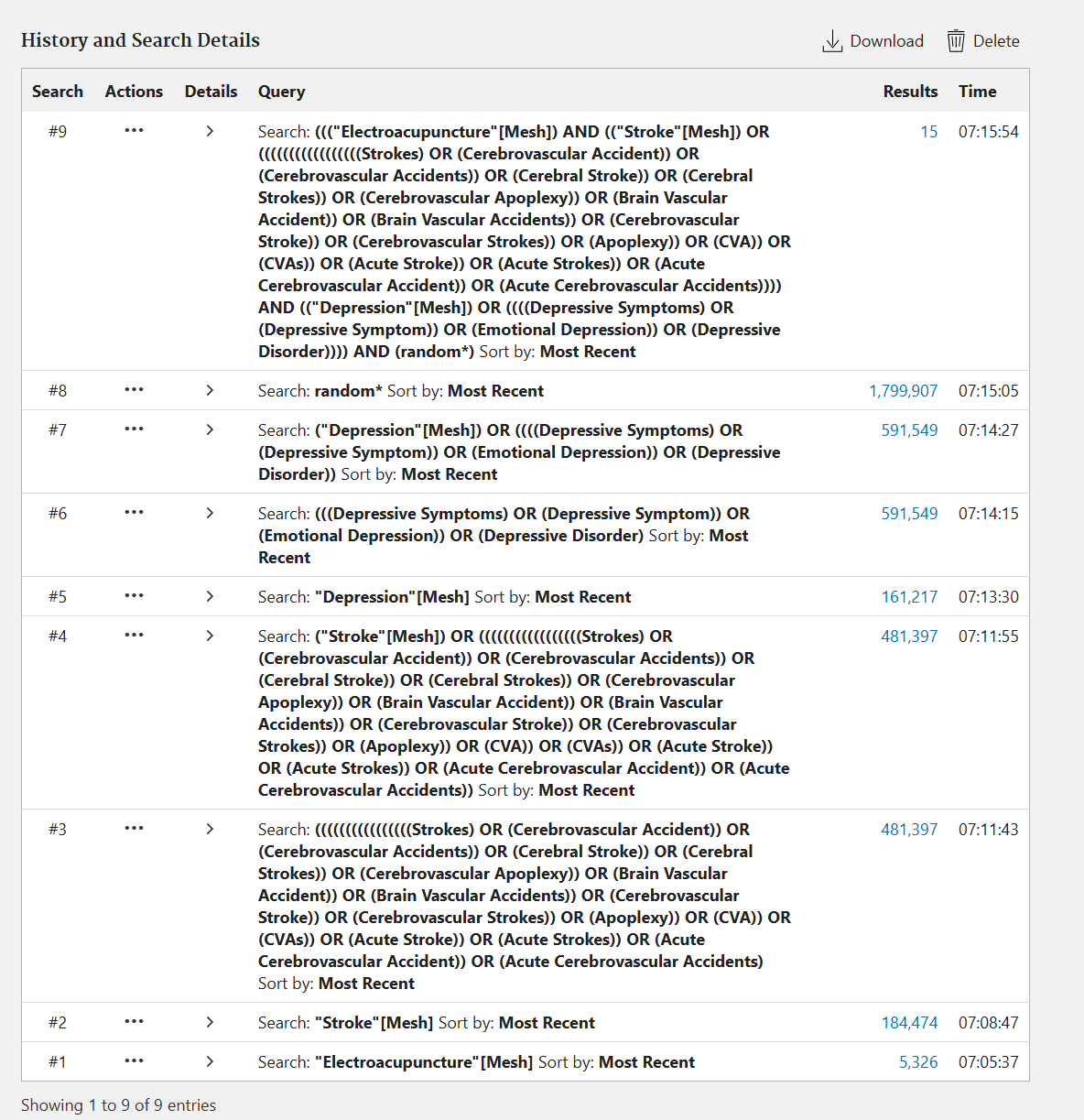


Embase-45

****(((Electroacupuncture) AND ((Stroke) OR (((((((((((((((((Strokes) OR (Cerebrovascular Accident)) OR (Cerebrovascular Accidents)) OR (Cerebral Stroke)) OR (Cerebral Strokes)) OR (Cerebrovascular Apoplexy)) OR (Brain Vascular Accident)) OR (Brain Vascular Accidents)) OR (Cerebrovascular Stroke)) OR (Cerebrovascular Strokes)) OR (Apoplexy)) OR (CVA)) OR (CVAs)) OR (Acute Stroke)) OR (Acute Strokes)) OR (Acute Cerebrovascular Accident)) OR (Acute Cerebrovascular Accidents)))) AND ((Depression) OR ((((Depressive Symptoms) OR (Depressive Symptom)) OR (Emotional Depression)) OR (Depressive Disorder)))) AND (random*)****


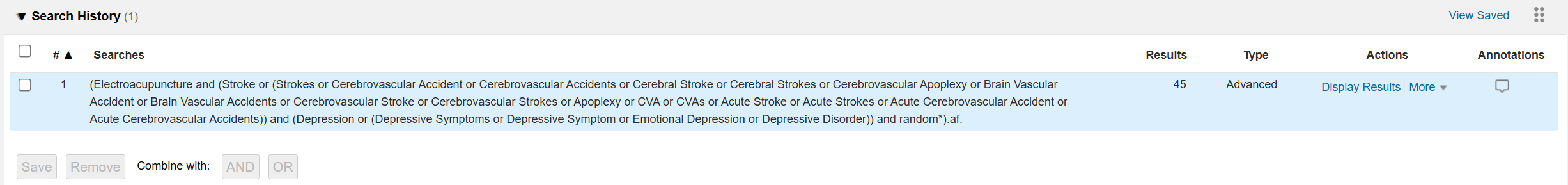
Cochrane-35

****(((Electroacupuncture) AND ((Stroke) OR (((((((((((((((((Strokes) OR (Cerebrovascular Accident)) OR (Cerebrovascular Accidents)) OR (Cerebral Stroke)) OR (Cerebral Strokes)) OR (Cerebrovascular Apoplexy)) OR (Brain Vascular Accident)) OR (Brain Vascular Accidents)) OR (Cerebrovascular Stroke)) OR (Cerebrovascular Strokes)) OR (Apoplexy)) OR (CVA)) OR (CVAs)) OR (Acute Stroke)) OR (Acute Strokes)) OR (Acute Cerebrovascular Accident)) OR (Acute Cerebrovascular Accidents)))) AND ((Depression) OR ((((Depressive Symptoms) OR (Depressive Symptom)) OR (Emotional Depression)) OR (Depressive Disorder)))) AND (random*)****


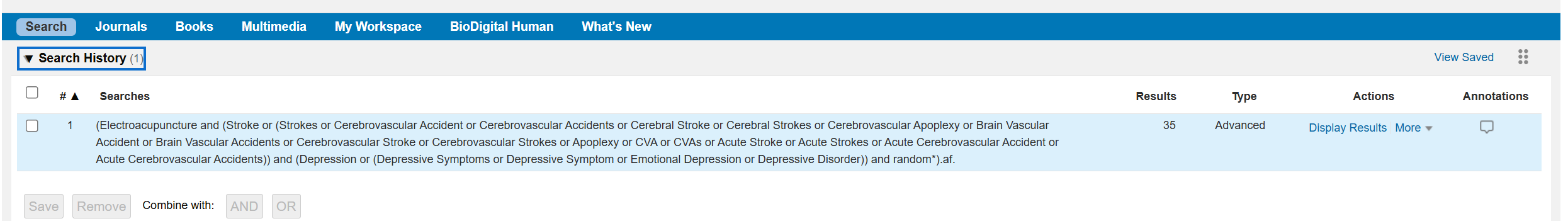


WOSS-28

**(((Electroacupuncture) AND ((Stroke) OR (((((((((((((((((Strokes) OR (Cerebrovascular Accident)) OR (Cerebrovascular Accidents)) OR (Cerebral Stroke)) OR (Cerebral Strokes)) OR (Cerebrovascular Apoplexy)) OR (Brain Vascular Accident)) OR (Brain Vascular Accidents)) OR (Cerebrovascular Stroke)) OR (Cerebrovascular Strokes)) OR (Apoplexy)) OR (CVA)) OR (CVAs)) OR (Acute Stroke)) OR (Acute Strokes)) OR (Acute Cerebrovascular Accident)) OR (Acute Cerebrovascular Accidents)))) AND ((Depression) OR ((((Depressive Symptoms) OR (Depressive Symptom)) OR (Emotional Depression)) OR (Depressive Disorder)))) AND (random*)** (topic)


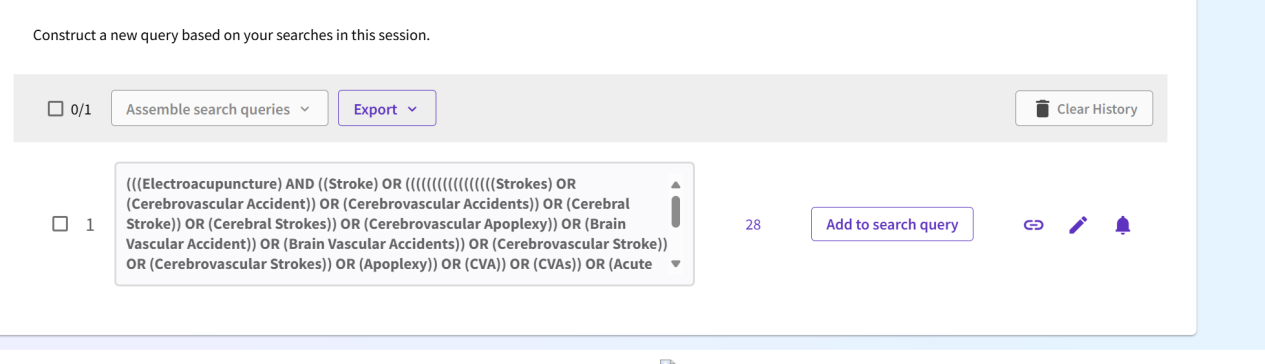


CNKI-215

（摘要：电针 + 电针灸(精确)）AND（摘要：卒中 + 脑血管意外 + 脑梗死 + 脑梗塞 + 脑卒中 + 脑血栓形成 +腔隙性脑梗死 + 脑出血 + 脑栓塞 + 蛛网膜下腔出血(精确)）AND（摘要：抑郁 + 抑郁症(精确)）


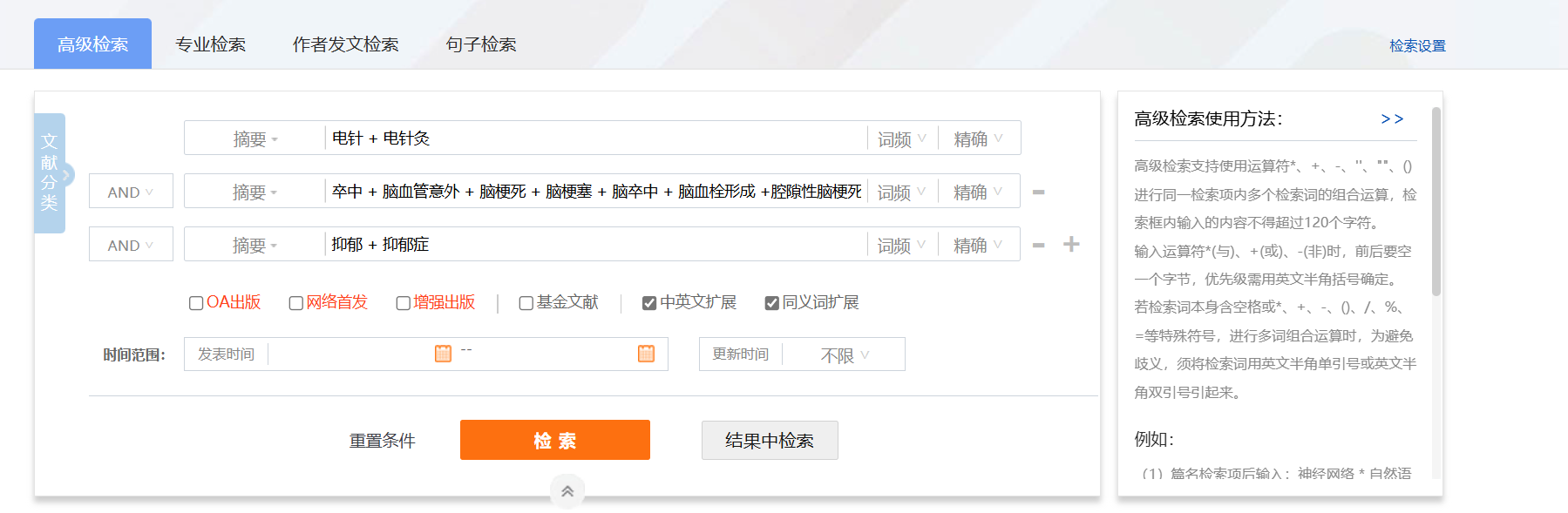


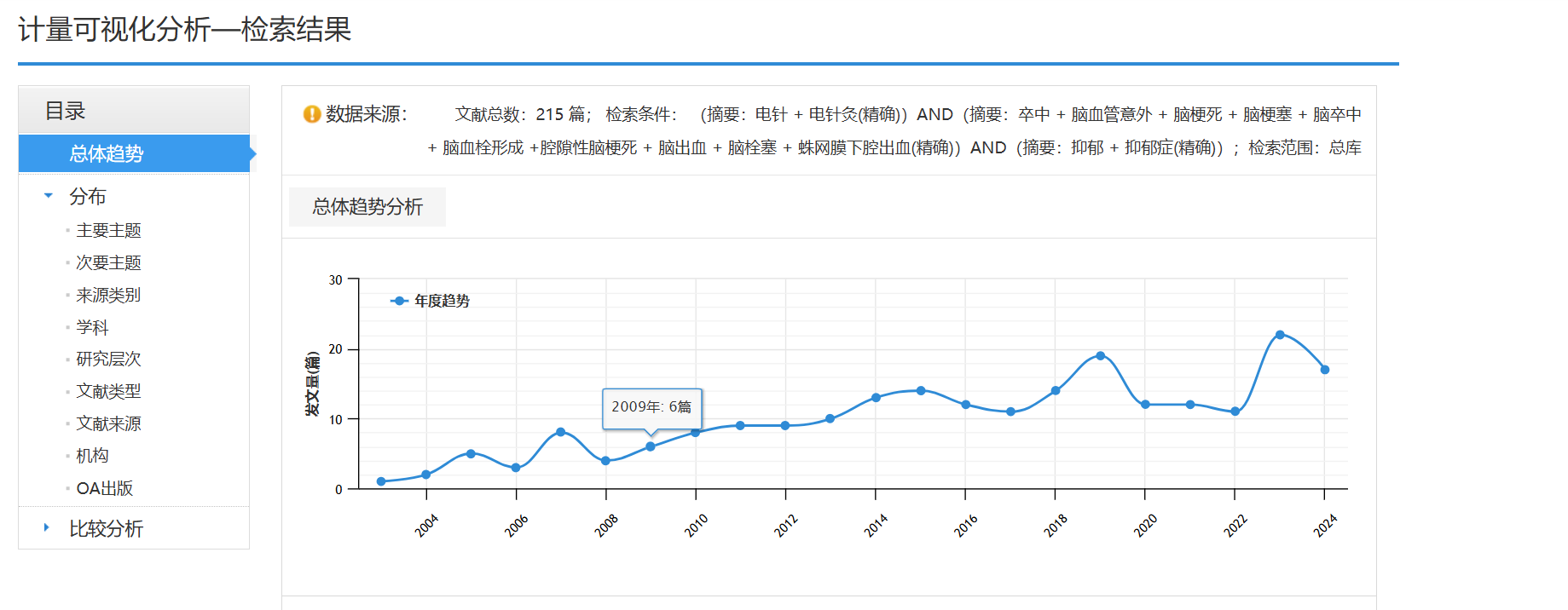


Wanfang-287

全部:(电针 OR 电针灸) and 全部:(卒中 OR 脑血管意外 OR 脑梗死 OR 脑梗塞 OR 脑卒中 OR 脑血栓形成 OR 腔隙性脑梗死 OR 脑出血 OR 脑栓塞 OR 蛛网膜下腔出血) and 全部:(抑郁 OR 抑郁症)


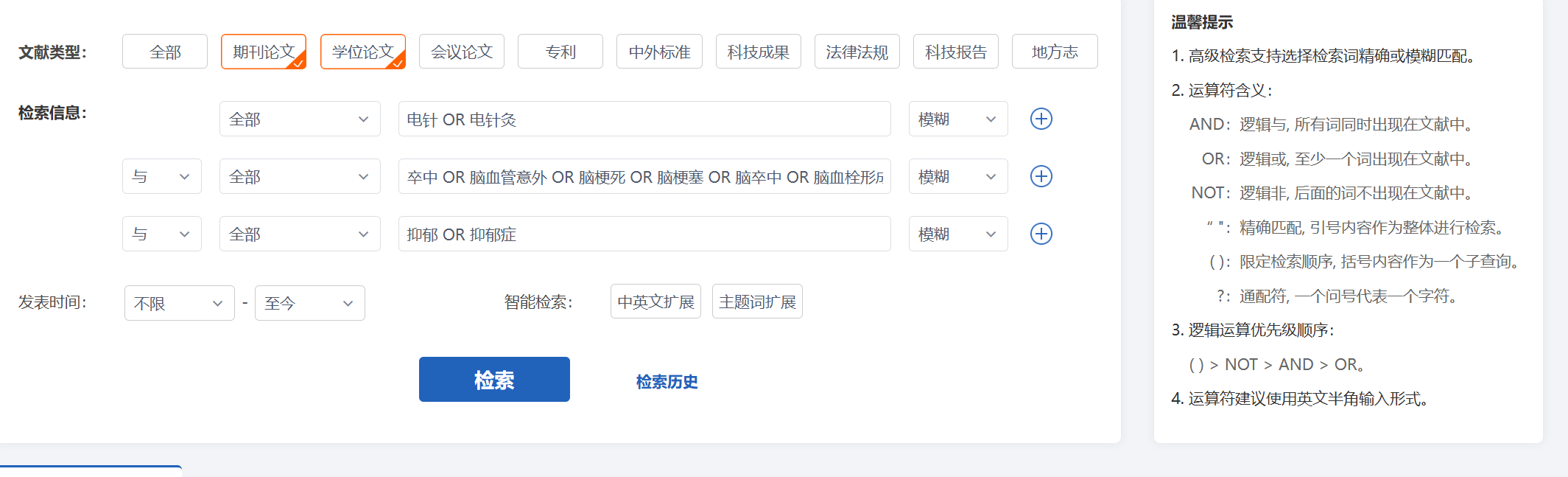

Supplement: Supplementary file 1 [file Table1.doc]
