# Supplementary material for: Efficacy and safety of electroacupuncture-based comprehensive treatment for post-stroke depression: a systematic review and meta-analysis of randomized controlled trials
Source: Front Psychiatry. 2025 Aug 15;16:1610032. doi: 10.3389/fpsyt.2025.1610032 (PMC12395381; doi:10.3389/fpsyt.2025.1610032)
Supplement: Supplementary file 2 [file Table2.doc]

Table S2 Clinical Efficacy Evaluation Criteria

| study | Diagnostic Criteria | cure rate | significant efficacy rate | effective rate | ineffectice rate |
| --- | --- | --- | --- | --- | --- |
| Cai 2019 | HAMD | 75%-100% | 50%-74% | 25%-49% | ＜25% |
| Cai 2020 | HAMD | 75%-100% | 50%-74% | 25%-49% | ＜25% |
| Chang 2011 | HAMD | 75%-100% | 50%-74% | 25%-49% | ＜25% |
| Cui 2008 | HAMD | 总分＜8 | ≥50% | 25%-49% | ＜25% |
| Deng 2016 | HAMD | 75%-100% | 50%-74% | 30%-49% | ＜30% |
| Ding 2020 | HAMD | 75%-100% | 50%-74% | 25%-49% | ＜25% |
| Ding 2023 | HAMD | 75%-100% | 50%-74% | 25%-49% | ＜25% |
| Dong 2007 I | HAMD | 75%-100% | 50%-74% | 25%-49% | ＜25% |
| Dong 2007 II | HAMD | 90%-100% | 50%-89% | 25%-49% | ＜25% |
| Dong 2007 II | SDS | ＜50% | 50%-59% | 60%-70% | ＞70% |
| Dong 2017 | HAMD | 90%-100% | 50%-89% | 25%-49% | ＜25% |
| Dong 2017 | SDS | ＜50% | 50%-59% | 60%-70% | ＞70% |
| Gao 2013 I | HAMD | 75%-100% | 50%-74% | 25%-49% | ＜25% |
| He 2020 | HAMD | 76%-100% | 50%-75% | 25%-49% | ＜25% |
| Hong 2015 | HAMD | 75%-100% | 50%-74% | 25%-49% | ＜25% |
| Huang 2014 | HAMD | 75%-100% | 50%-74% | 25%-49% | ＜25% |
| Jiang 2017 | HAMD | 85%-100% | 50%-84% | 25%-49% | ＜25% |
| Li 2013 | HAMD | 75%-100% | 50%-74% | 25%-49% | ＜25% |
| Liu 2015 I | HAMD | 75%-100% | 50%-74% | 25%-49% | ＜25% |
| Liu 2015 II | HAMD | 75%-100% | 50%-74% | 25%-49% | ＜25% |
| Meng 2023 | HAMD | 90%-100% | 65%-89% | 30%-64% | ＜30% |
| Ni 2023 | HAMD | 76%-100% | 50%-75% | 25%-49% | ＜25% |
| Shao 2020 | HAMD | 75%-100% | 50%-70% | 25%-49% | ＜25% |
| Wang 2015 | HAMD | 91%-100% | 46%-90% | 18%-45% | ＜17% |
| Wang 2016 | HAMD | 75%-100% | 51%-74% | 25%-50% | ＜25% |
| Wang 2020 | HAMD | 75%-100% | 50%-74% | 25%-49% | ＜25% |
| Wang 2023 | HAMD | 75%-100% | 50%-74% | 25%-49% | ＜25% |
| Xu 2014 | HAMD | 76%-100% | 50%-75% | 25%-49% | ＜25% |
| Yang 2013 | HAMD | ＜7分 | ≥50% | 30%-49% | ＜30% |
| You 2013 | HAMD | 76%-100% | 50%-75% | 25%-49% | ＜25% |
| Yu 2013 | HAMD | 75%-100% | 50%-74% | 25%-49% | ＜25% |
| Zhang 2016 | HAMD | 75%-100% | 50%-74% | 25%-49% | ＜25% |
| Zhang 2017 | HAMD | 75%-100% | 50%-74% | 25%-49% | ＜25% |
| Zuo 2023 | HAMD | 75%-100% | 50%-74% | 25%-49% | ＜25% |
