# Supplementary material for: Efficacy and safety of electroacupuncture-based comprehensive treatment for post-stroke depression: a systematic review and meta-analysis of randomized controlled trials
Source: Front Psychiatry. 2025 Aug 15;16:1610032. doi: 10.3389/fpsyt.2025.1610032 (PMC12395381; doi:10.3389/fpsyt.2025.1610032)
Supplement: Supplementary file 3 [file Table3.doc]

Table S3 Research characteristics

| **Study** | **study period (study period)** | **region (region)** | **study design(type of research)** | **Population (target population)** | **Diagnostic criteria** | **Intervention group** | **Control group** |
| --- | --- | --- | --- | --- | --- | --- | --- |
|
| Bi 2009a | 2005.7-2008.9 | China | RCT | Consistent with Chinese and Western medicine diagnosis of stroke, confirmed by CT and MRI; No obvious loss of intelligence and aphasia; No positive personal and family history of mental disorders, no recent use of antidepressants; Agreed to be enrolled | HAMD/SDS | Electroacupuncture Baihui（GV20）, Yintang（GV24）, anti-depression waveform, 2 ~ 3V, 45min/time, once/d, 5 times a week | Rehabilitation therapy, the main use of nerve promoting technology to treat ‚ such as Bobath therapy, neuroproprioceptive promoting technology, motor relearning technology and so on |
| Bi 2009b | 2005.7-2008.9 | China | RCT | Consistent with Chinese and Western medicine diagnosis of stroke, confirmed by CT and MRI; No obvious loss of intelligence and aphasia; No positive personal and family history of mental disorders, no recent use of antidepressants; Agreed to be enrolled | HAMD/SDS | Electroacupuncture Baihui（GV20）, Yintang（GV24）, antidepressant waveform, 2 ~ 3V, 45min/time, once /d, 5 times a week + fluoxetine 20mg/d | Rehabilitation therapy, the main use of nerve promoting technology to treat  such as Bobath therapy, neuroproprioceptive promoting technology, motor relearning technology and so on |
| Bi 2009c | 2005.7-2008.9 | China | RCT | Consistent with Chinese and Western medicine diagnosis of stroke, confirmed by CT and MRI; No obvious loss of intelligence and aphasia; No positive personal and family history of mental disorders, no recent use of antidepressants; Agreed to be enrolled | HAMD/SDS | Electroacupuncture Baihui（GV20）, Yintang（GV24）, anti-depression waveform, 2 ~ 3V, 45min/time, 1 time/day, 5 times a week + rehabilitation treatment | Rehabilitation therapy, the main use of nerve promoting technology to treat  such as Bobath therapy, neuroproprioceptive promoting technology, motor relearning technology and so on |
| Bi 2009d | 2005.7-2008.9 | China | RCT | Consistent with Chinese and Western medicine diagnosis of stroke, confirmed by CT and MRI; No obvious loss of intelligence and aphasia; No positive personal and family history of mental disorders, no recent use of antidepressants; Agreed to be enrolled | HAMD/SDS | Electroacupuncture Baihui（GV20）, Yintang（GV24）, anti-depression waveform, 2 ~ 3V, 45min/time, 1 time/day, 5 times a week + fluoxetine 20mg/d+ rehabilitation | Rehabilitation therapy, the main use of nerve promoting technology to treat  such as Bobath therapy, neuroproprioceptive promoting technology, motor relearning technology and so on |
| Cai 2019 | 2017.11-2019.3 | China | RCT | The patients diagnosed with cerebral infarction or cerebral hemorrhage are between 40 and 70 years old, regardless of gender; No other antidepressant therapy was used and they agreed to be enrolled | HAMD 8≤ Total ≤35 | Electroacupuncture Baihui（GV20）, Taichong（LR3）, Sanyinjiao(SP6), GanShu(BL18) + fluoxetine 20mg/d | Fluoxetine 20mg/d |
| Cai 2020a | 2019.3-2020.01 | China | RCT | CT, MRI confirmed cerebral infarction or cerebral hemorrhage patients, the course of disease ≥2 weeks; Age between 40 and 75 years old, gender is not limited; Be conscious; Did not use other antidepressant treatment and agreed to be enrolled | 24 HAMD scores ≥8 points, < 35 points | Electroacupuncture Baihui（GV20）, Shenting (DU24), Shenmen(HT7) (double), Neiguan(PC6) (double), Taichong（LR3） (double) acupoints, Shu-wave, 5HZ, depth 0.5-0.8 inches + basic drug treatment | Dan Shu(BL19) (double), Ge Shu(BL17) (double) moxibustion + basic drug treatment |
| Cai 2020b | 2019.3-2020.01 | China | RCT | CT, MRI confirmed cerebral infarction or cerebral hemorrhage patients, the course of disease ≥2 weeks; Age between 40 and 75 years old, gender is not limited; Be conscious; Did not use other antidepressant treatment and agreed to be enrolled | 24 HAMD scores ≥8 points, < 35 points | Electroacupuncture Baihui（GV20）, Shenting (DU24), Shenmen(HT7) (double), Neiguan(PC6) (double), Taichong（LR3） (double) acupoints, Shubo, 5HZ,+ wheat moxibustion Sihua point, Dan Shu (double), Geshu (double) + basic drug treatment | Dan Shu(BL19) (double), Ge Shu(BL17) (double) moxibustion + basic drug treatment |
| Chang 2011 | 2007.5-2009.5 | China | RCT | Patients with various cerebrovascular diseases confirmed by CT and MRI; HAMD > 8; First onset, duration ≥3 weeks, depressive symptoms ≥2 weeks; Age 40-75 years old | HAMDScale/Self-rating SDS Scale for depression | Electroacupuncture Baihui（GV20）, Shenting (DU24), Neiguan(PC6), Shenmen(HT7), Sishencong, Hegu（LI4）, Taichong（LR3）, Sanyinjiao(SP6) point; Dredging wave; 30min/ time, once/day, 6 times/week + oral fluoxetine hydrochloride capsule (Prozac) once/day | Oral fluoxetine hydrochloride capsules (Prozac) once /d for 28 days |
| Chen 2012 | 2011.6-2011.9 | China | RCT | For patients with post-stroke depression; A medical history of more than 3 months; And depression for more than two weeks | HAMD Scale | Rehabilitation + electroacupuncture Baihui（GV20）, Fengchi(GB20), Hegu（LI4） electroacupuncture treatment; 1HZ, density wave, stimulation for patient tolerance; 30min/ time, 3 times/week | Take fluoxetine 20mg/d and recover |
| Chen 2014 | 2012.10-2013.10 | China | RCT | CT or MRI results were positive for cerebral hemorrhage or cerebral infarction; No prior organic disease; HAMD≥16, SDS≥50%; Age 30-years old | HAMD/SDS | Electric acupuncture Neiguan(PC6), Shenmen(HT7), Sanyin Jiao(SP6), Hegu（LI4）, Taichong（LR3）, 200r/min, frequency of 120-250 times /min, 1 time /d, 10 times/course, 3 courses of treatment | Electroacupuncture Baihui（GV20）, Yintang（GV24）, Sishencong, Neiguan(PC6), Sanyinjiao(SP6), Hegu（LI4）, Taichong（LR3）, 30min/ time, 10 times/course, 3 courses of treatment |
| Cheng 2007Ⅰa | \ | China | RCT | Met diagnostic criteria for stroke and depression; Onset > 2 weeks; HAMD > 20, SDS > 41; No history of mental illness and alcohol abuse; And signed informed consent | HAMD/SDS | Electroacupuncture Baihui（GV20）, Shenting (DU24), Yintang（GV24）, Sishencong, Taichong（LR3）, Shenmen(HT7), voltage is 6V, density wave; The strength was patient tolerance, the retention needle was energized for 30min, once /d, a total of 21 times, 6 weeks | Non-current abdominal acupuncture was used for 30min, once /d, for 6 weeks |
| Cheng 2007Ⅰb | \ | China | RCT | Met diagnostic criteria for stroke and depression; Onset > 2 weeks; HAMD > 20, SDS > 41; No history of mental illness and alcohol abuse; And signed informed consent | HAMD/SDS | Electroacupuncture Baihui（GV20）, Shenting (DU24), Yintang（GV24）, Sishencong, Taichong（LR3）, Shenmen(HT7), voltage is 6V, density wave; The strength was patient tolerance, the retention needle was energized for 30min, once /d, a total of 21 times, 6 weeks | Rehabilitation |
| Cheng 2007Ⅱ a | 2006.3-2007. | China | RCT | Meet the diagnostic criteria of stroke depressive episode; Age 60-85 years old, onset time > 2 weeks; 20 < HAMD < 35; SDS > 41; No previous psychosis and substance abuse, no drug allergies, agree to cooperate with the examination | HAMD Scale | Electric acupuncture Baihui（GV20）, Shenting (DU24), Yintang（GV24） Sishencong, Taichong（LR3）, Shenmen(HT7), voltage is 6V, density wave; The strength was patient tolerance, the retention needle was energized for 30min, once /d, a total of 21 times, 6 weeks | Basic drug therapy |
| Cheng 2007Ⅱ b | 2006.3-2007. | China | RCT | Meet the diagnostic criteria of stroke depressive episode; Age 60-85 years old, onset time > 2 weeks; 20 < HAMD < 35; SDS > 41; No previous psychosis and substance abuse, no drug allergies, agree to cooperate with the examination | HAMD Scale | Electric acupuncture Baihui（GV20）, Shenting (DU24), Yintang（GV24） Sishencong, Taichong（LR3）, Shenmen(HT7), voltage is 6V, density wave; The strength was patient tolerance, the retention needle was energized for 30min, once /d, a total of 21 times, 6 weeks | The non-current abdominal acupuncture group was retained for 30Min, once /d, a total of 21 times |
| Cui 2008 | 2006.52008.4 | China | RCT | The diagnosis of stroke was confirmed by CT or MR; HAMD > 7; Age < 85, duration of disease < 2 years; Patients receiving treatment | HAMD Scale | Electroacupuncture Baihui（GV20）, Shenmen(HT7), Shenting (DU24), plus electric, thin wave, 5HZ; 30min, 5 times/week, 6 weeks in total | Non-current electroacupuncture therapy |
| Deng 2016 | 2014.1-2014.12 | China | RCT | Diagnosed with PSD after CT or MR; 52 > HAMD > 7; Ages 50-85; The course of disease ≤6 months liver qi stagnation, heart and spleen deficiency | HAMD Scale | Rehabilitation + electroacupuncture Taichong（LR3）, Neiguan(PC6) or Gongsun(SP4), Neiguan(PC6); 30min/ time, Shubo, 5HZ, 5 times/week for 6 weeks | Rehabilitation |
| Ding 2020 | 2017.2-2019.1 | China | RCT | For patients with stroke depression; HAMD > 18; Ages 35-75; Signed informed consent form | HAMD Scale | Oral fluoxetine hydrochloride capsule (Prozac) 2 times /d+ electric acupuncture Neiguan(PC6), Shenmen(HT7), Hegu（LI4）, Sanyinjiao(SP6), Sishencong, 30Min/ time, 6 times/week for 4 weeks | Oral fluoxetine hydrochloride capsule (Prozac) twice /d for 4 weeks |
| Ding 2023a | 2020.1-2022.12 | China | RCT | Meet the diagnostic criteria of Western Medicine; Age 40-75 years old; The duration of stroke was within 1 year after the acute stage of stroke; Depression was the first occurrence caused by stroke; Volunteer to join the study | HAMD Scale | Electroacupuncture Baihui（GV20）, Shenting (DU24), electrification, continuous wave, 60HZ, retention needle 30min, once /d | Oral edbenquinone tablet 30mg/ tablet, 2 tablets /d after meals |
| Ding 2023b | 2020.1-2022.12 | China | RCT | Meet the diagnostic criteria of Western Medicine; Age 40-75 years old; The duration of stroke was within 1 year after the acute stage of stroke; Depression was the first occurrence caused by stroke; Volunteer to join the study | HAMD Scale | Baihui（GV20）, Shenting (DU24), electrification, continuous wave, 60HZ, retaining needle for 30min, 1 time /d+ Siqi decoction (1 dose /d, 150ml/ time, 3 times /d, oral) | Oral edbenquinone tablet 30mg/ tablet, 2 tablets /d after meals |
| Dong 2007Ⅰ | 2003.6-2005.6 | China | RCT | Diagnosed with cerebral infarction or cerebral hemorrhage by CT or MR; First onset within 1 month; No previous medical history | HAMD Scale | Rehabilitation +, electroacupuncture Baihui（GV20）, Shenting (DU24), Neiguan(PC6), Shenmen(HT7), Sanyinjiao(SP6), Taichong（LR3）, density wave, frequency 1-2HZ; 30min/ time, 1 time/day, 6 times/week; At the same time, oral prozac 20mg, once /d | Oral Prozac 20mg, 1 dose/day plus recovery |
| Dong 2007Ⅱa | \ | China | RCT | In line with the diagnostic criteria of stroke depression, cerebral hemorrhage and cerebral infarction occurred for the first time, and CT or MR Was positive; No previous history, time > 2 weeks; HAMD > 20, SDS > 60%; Ages 31-70 | HAMD Scale/SDS | Electroacupuncture Xuanlu(GB5), xuan Li(GB6), Naohu(GV15), Qiangjian(ST3), Tou Linqi(GB1), Yang Bai(GB14), shuai gu(SJ5), Qubin(GB6), Shenting (DU24), Yintang（GV24）, continuous wave, frequency 120 ~ 250 times per minute power 30min/ time, retention needle 1h, 1 time /d, 10 times/course, a total of 3 courses | No current electroacupuncture Baihui（GV20）, Yintang（GV24）, Sishencong, Neiguan(PC6), Shenmen(HT7), Sanyinjiao(SP6), Hegu（LI4）, Taichong（LR3）, retained needle 1h, 1 time /d, 10 times/course, a total of 3 courses |
| Dong 2007Ⅱb | \ | China | RCT | In line with the diagnostic criteria of stroke depression, cerebral hemorrhage and cerebral infarction occurred for the first time, and CT or MR Was positive; No previous history, time > 2 weeks; HAMD > 20, SDS > 60%; Ages 31-70 | HAMD Scale/SDS | Electroacupuncture Xuanlu(GB5), xuan Li(GB6), Naohu(GV15), Qiangjian(ST3), Tou Linqi(GB1), Yang Bai(GB14), shuai gu(SJ5), Qubin(GB6), Shenting (DU24), Yintang（GV24）, continuous wave, frequency 120 ~ 250 times per minute power 30min/ time, retention needle 1h, 1 time /d, 10 times/course, a total of 3 courses | Western medicine oral fluoxetine in doses from 20mg/ day to 80mh/ day |
| Dong 2016a | 2010.2-2011.2 | China | RCT | Patients with post-stroke depression | HAMD Scale | Electroacupuncture Yintang（GV24）, Baihui（GV20）, Hegu（LI4）, Taichong（LR3）, Shenting (DU24), Sishencong; Dense wave. With patient tolerance as the limit, needle retention 30min, once a day, 5 days/course, a total of 6 courses | Drug treatment (Escitalopram) normal dose 20mg, severe patients 40mg, once a day, 7 days/course |
| Dong 2016b | 2010.2-2011.2 | China | RCT | Patients with post-stroke depression | HAMD Scale | Electric Needle Yintang（GV24）, Baihui（GV20）, Hegu（LI4）, Taichong（LR3）, Shenting (DU24), Sishencong; Dense wave. With patient tolerance as the limit, keep the needle for 30min, once a day, 5 days/course + Esitalopram | Drug treatment (Escitalopram) normal dose 20mg, severe patients 40mg, once a day, 7 days/course |
| Dong 2017a | 2013.1-2015.6 | China | RCT | For the first cerebral infarction or cerebral hemorrhage and the CT or MR Test is positive; Clear consciousness; HAMD > 20, SDS > 60% age 31-70 years; Informed consent | HAMD Scale/SDS | Electric acupuncture Shenmen(HT7), Sanyinjiao(SP6), Neiguan(PC6), Hegu（LI4）, Taichong（LR3）, energize 30min, once /d, 10 times/course of treatment, a total of 3 courses | Western medicine oral fluoxetine hydrochloride, the dose gradually increased from 20mg/d to 80mg/d |
| Dong 2017b | 2013.1-2015.6 | China | RCT | For the first cerebral infarction or cerebral hemorrhage and the CT or MR Test is positive; Clear consciousness; HAMD > 20, SDS > 60% age 31-70 years; Informed consent | HAMD Scale/SDS | Electric acupuncture Shenmen(HT7), Sanyinjiao(SP6), Neiguan(PC6), Hegu（LI4）, Taichong（LR3）, energized 30min, once /d, 0 times/course, a total of 3 courses | Ordinary electroacupuncture without current |
| Feng 2012 | 2008.7-2011.12 | China | RCT | Met the diagnostic criteria for stroke, confirmed by CT or MR, nerve injury > 7; HAMD0 scores of 20-35; Ages 30-70; Alert and willing to cooperate with research; Not taking other medications for more than 2 weeks | HAMD Scale/SDS | Electroacupuncture Baihui（GV20）, Shenting (DU24), Yintang（GV24）, Yangbai(GB14), Sishencong, TaiYang, Neiguan(PC6), Hegu（LI4）, Taichong（LR3）, Fenglong (ST40), Sanyinjiao(SP6); Density wave, frequency 1hz, retention needle 30min, 1 time /d, 5 times/week; Oral Xingnaojieyu capsule, 3 capsules/time, 3 times /d | False acupuncture + oral Delisin, 1 tablet/time, 2 times /d |
| Gao 2013 | 2011.1-2012.7 | China | RCT | It met the diagnostic standards of Chinese and Western medicine and was confirmed by CT or MR Test; Drug withdrawal for more than two weeks; HAMD > 8, < 17; Age 45-75 years, duration of disease < 2 years; Clear awareness to cooperate with treatment, informed and consenting to research | HAMD Scale | Rehabilitation + electroacupuncture Baihui（GV20）, Sishencong, Shenting (DU24), Benshen(GV16), Yintang（GV24）, Yangbai(GB14), electrification, density wave, retention needle 30min, twice /d,6d/ week, a total of 8 weeks, oral fluoxetine hydrochloride capsule | Oral fluoxetine hydrochloride capsule 20mg/ time, 2 times /d+ rehabilitation |
| Gao 2013‖ | 2011.7-2013.2 | China | RCT | Age 40-70; Eligible stroke patients; Depressive symptoms 2 weeks after stroke onset; HAMD≥8, < 35; No previous history of mental disorders; Signed informed consent form | HAMD Scale | Rehabilitation + electroacupuncture Baihui（GV20）, Zusanli(ST36), continuous wave, retention of needle 30min, once /d, a total of 4 weeks; Take the drug Zoloft, normal 50mg once a day, poor efficacy twice a day | Oral medication Zoloft + rehabilitation |
| Guo 2011a | 2007. - 509.5 | China | RCT | For patients with stroke depression, verified by CT or MRI, the diagnosis of depression is consistent Criteria in the Chinese Classification and Diagnostic Criteria for Mental Disorders (CCMD-3) Quasi, depression score 13 to 32 points | HAMD Scale | Electroacupuncture Baihui（GV20）, Shenting (DU24), Yintang（GV24）, Sishencong, Taichong（LR3）, Hegu（LI4）, Dredging wave; The strength was patient tolerance, the retention needle was energized for 30min, once /d, 5 times/week | Fluoxetine was taken orally 20mg once per day for 6 weeks |
| Guo 2011b | 2007. - 509.5 | China | RCT | For patients with stroke depression, verified by CT or MRI, the diagnosis of depression is consistent Criteria in the Chinese Classification and Diagnostic Criteria for Mental Disorders (CCMD-3) Quasi, depression score 13 to 32 points | HAMD Scale | Electric injection of Baihui（GV20）, Shenting (DU24), Yintang（GV24）, Sishencong, Hegu（LI4）, Taichong（LR3）, Shumibo 30min+ oral fluoxetine 20mg | Fluoxetine was taken orally 20mg once per day for 6 weeks |
| He 2020 | 2018.3-1019.6 | China | RCT | For stroke patients, HAMD≥7, HAMA > 8; The patient's condition was basically stable, and informed consent was signed | HAMD Scale | Rehabilitation, electroacupuncture Baihui（GV20）, Sishencong, TaiYang, Shuigou(ST9), 30min/ time, 5 times/week, treatment for 2 weeks; And electroacupuncture on both sides of Taichong（LR3）, Zusanli(ST36), Neiguan(PC6), once /d, treatment for 2 weeks; Low-frequency repetitive transcranial magnetic stimulation, once d, 30min, 5 times/week, | Rehabilitation |
| Hong 2015 | 2013.1-2014.1 | China | RCT | First onset, stroke diagnosis criteria, duration > 2 weeks; HAMD≥18, < 35; Age 35-80 years old; Conscious, cooperative, willing to sign informed consent | HAMD Scale | Rehabilitation + electroacupuncture Baihui（GV20）, Sishencong, Shenting (DU24), Zusanli(ST36), Sanyin Jiao(SP6), Taichong（LR3）, keep the needle for 30min, 6 times/week | Oral Citalopram tablet 20mg, once /d, 1 tablet/time for 30 consecutive days + rehabilitation |
| Huang 2005 | 2001.5-2002.8 | China | RCT | Diagnosed by CT or MRI, eligible for depression; HAMD > 20 | HAMD Scale | Scalp electroacupuncture top midline, frontal midline and posterior frontal line, energize and retain needle for 30min, 6 times/week | No current acupuncture treatment |
| Huang 2014a | 2011.3-2012.12 | China | RCT | Meet the diagnostic criteria for stroke and post-stroke depression; HAMD > 7; Age 40-75 years, disease course 2 years younger; Volunteer for study | HAMD Scale | Electroacupuncture Baihui（GV20）, Qiangjian(ST3), Benshen(GV16), Tianchong(GB16), electrification, with density wave, frequency 2-100 Hz, intensity 0. Current of 1-1 mA, 30min, 5 times/week | Fluoxetine was taken orally 20mg once per day for 6 weeks |
| Huang 2014b | 2011.3-2012.12 | China | RCT | Meet the diagnostic criteria for stroke and post-stroke depression; HAMD > 7; Age 40-75 years, disease course 2 years younger; Volunteer for study | HAMD Scale | Electric needle Baihui（GV20）, Qiangjian(ST3), Benshen(GV16), Tianchong(GB16), power, with density wave, frequency 2-100 Hz, strength 0. 1-1mA current, 30min, 5 times/week + oral drug fluoxetine 20mg | Fluoxetine was taken orally 20mg once per day for 6 weeks |
| Huang 2019 | 2018.3-2019.3 | China | RCT | The patient was diagnosed with stroke; Enrolled within 2 weeks of onset; Patients participated voluntarily; | HAMD Scale/SDS | Fluoxetine hydrochloride + upper head electroacupuncture treatment, electroacupuncture Yintang（GV24）, Yang Bai(GB14), etc., 30min; The intensity was patient tolerance, keep the needle for 1h, 1 time/day, 10 times/course | Fluoxetine hydrochloride therapy + acupuncture without current |
| Jiang 2006 | 2003.2-2005.11 | China | RCT | Meet the diagnostic criteria of cerebrovascular disease and cerebral infarction; Meet the diagnostic criteria of depression disorder; And rule out psychiatric disorders, etc | HAMD Scale | Electroacupuncture Baihui（GV20）, Fengchi(GB20), Shenting (DU24), Neiguan(PC6) and Shenmen(HT7) electroacupuncture treatment; 1HZ, density wave, stimulation for patient tolerance; 1 time/day, 20min/ time + oral fluoxetine hydrochloride 20mg | Oral fluoxetine hydrochloride |
| Jiang 2017 | \ | China | RCT | For patients with post-stroke depression; Meet the diagnostic criteria of cerebral hemorrhage and cerebral infarction; HAMD score 8-35; Sign informed consent form | HAMD Scale | Electroacupuncture Baihui（GV20）, Yintang（GV24）, density wave /100hz, lasting 30min, 1 time /d, 5 days/course, a total of 4 courses | Oral Prozac 20mg, once /d |
| Jiao 2018 | 2016.6-2018.2 | China | RCT | Meet the diagnostic criteria of acute cerebrovascular disease, confirmed by CT or MRI; HAMD at 21-35; And no history of mental illness | HAMD Scale | Oral drug fluoxetine hydrochloride capsule + electroacupuncture Shenmen(HT7), heart and ear point electrify 30min, once /d, 5 times/week | Oral drug fluoxetine hydrochloride capsule 20mg once /d |
| Kang 2014 | 2012.3-2014.3 | China | RCT | Diagnosed as cerebral apoplexy, first onset; The course of the disease was within 3 months; Meet the diagnostic criteria of post-stroke depression; HAMD > 20; Clear consciousness with treatment; And be 18-80 years old | HAMD Scale | Basic treatment of neurology plus electroacupuncture Shenting (DU24), Fengchi(GB20), etc., electrification and density wave, the current size of the patient's tolerance 30min, once /d | Oral fluoxetine hydrochloride 20mg was added for basic treatment of neurology |
| Li 2013 | 2011.4-2012.4 | China | RCT | In line with the diagnostic points of various cerebrovascular diseases: stroke confirmed by CT and MRI, duration ≥2 weeks; clear consciousness, no cognitive impairment or language impairment; depression diagnosis in line with the therapeutic Criteria for Diagnosis of TCM Diseases and Syndrome · Depression | Total score of HAMD ≥ 8 | Electroacupuncture Sishencong, Shenting (DU24), Benshen(GV16), Hegu（LI4） (bilateral), Taichong（LR3） (bilateral) + basic treatment of neurology | Fluoxetine hydrochloride 20mg/d+ basic treatment in neurology |
| Liu 2015 Ⅰ | 2009.7-2012.6 | China | RCT | In line with Western medicine cerebral hemorrhage, cerebral infarction, depression; In line with TCM stroke, depression syndrome standards; Age 40 ~ 75 years old; Cooperate with treatment, the diagnosis of depression conforms to the "TCM disease diagnosis and curative effect standard · Depression disease" | HAMD total score ≥ 17 | Electroacupuncture treatment of Neiguan(PC6), Shenting (DU24), Hegu（LI4）, Shenmen(HT7), Baihui（GV20）, Sishencong, Sanyin Jiao(SP6), Taichong（LR3） (both double), Qimen(LV14) + Chinese medicine with Bupleurum Shugan SAN | Fluoxetine 20mg/d |
| Liu 2015 Ⅱ | \ | China | RCT | Cerebral apoplexy occurred for the first time, which was confirmed by CT and MRI, in line with the diagnostic criteria of traditional Chinese and Western medicine; 40 years old < 75 years old, duration ≥2 weeks; Agreed to be enrolled | HAMD 18 < Total score < 35 | Chinese medicine plus electroacupuncture Taichong（LR3）, Hegu（LI4）, Baihui（GV20）, Yintang（GV24）, Neiguan(PC6), Shenmen(HT7) (both double). Denser wave; 30min/ time, 3 times/week for 8 consecutive weeks | Flupentixol 0.5mg/ time, melitracen 10mg/ time, 2 times/day |
| Liu 2016 | 2012.2-2015.3 | China | RCT | CT and MRI confirmed stroke, no obvious limb paralysis course ≥3 months; Lucid and agreed to be enrolled | HAMD 24 scores ≥17 | Delisin 0.5 mg, 2 times/day; Electroacupuncture Baihui（GV20）, Yintang（GV24）, Shanzhong(RN17), Neiguan(PC6) (double), Shen Men (double), Taichong（LR3） (double), 30 min/ time, 1 time/day, 6 times/course | Deisin 0.5 mg, 2 times /d |
| Liu 2021 | 2018.1-2018.12 | China | RCT | In line with the diagnostic points of various cerebrovascular diseases, stroke confirmed by CT and MRI, disease course 1 ~ 180 days; non-taking antidepressants; motor aphasia; agreed to be included in the group | Depression Questionnaire for Aphasia after Stroke (SADQ-H) | Electroacupuncture at Fengchi(GB20) point and blood supply point, 30min/ time, continuous treatment for 6 times, rest for 1 day,1 course in 1 month, continuous treatment for 2 courses + conventional treatment in neurology | Fluoxetine hydrochloride 20mg/d+ basic treatment in neurology |
| Long 2004 | 2002.6-2003.6 | China | RCT | CT and MRI confirmed that the stroke patient had unconsciousness disorder | HAMD Scale | Electroacupuncture Baihui（GV20）, Yintang（GV24）, TiWaiGuan(SJ5), Hegu（LI4）, Taichong（LR3）, Zusanli(ST36), 2Hz, 2V, density wave, once /d, 45min/ times, 6 times/week + routine treatment in neurology department | Fluoxetine 10~40mg/d+ routine treatment in neurology department |
| Meng 2023 | 2021.12-2023.1 | China | RCT | In line with traditional Chinese and Western medicine diagnosis of stroke, post-stroke depression; Age ≥60 years old; Not taking psychotropic drugs and agree to be enrolled | HAMD score ≥17 | Repeated transcranial magnetic stimulation (rT⁃MS) treatment combined with electroacupuncture Baihui（GV20） and Sishencong; Bilateral Benshen(GV16); Affected side quchi(LI11), Neiguan(PC6); Zu Sanli(ST36), Shen Mai(DU14), density wave, 1Hz, 30min/ time, 5 times/week | Repeat transcranial magnetic stimulation (rT⁃ MS) treatment |
| Ni 2023 | 2020.1-2022.1 | China | RCT | Patients with post-stroke depression, duration ≥1 week; Age 40 ~ 80 years old; No personal or family history of mental disorders; Consent to inclusion | HAMD score ≥17 | Sertraline hydrochloride tablets 20 mg/d; Electroacupuncture Baihui（GV20）, Sishencong, Yintang（GV24）, Neiguan(PC6), Hegu（LI4）, Zusanli(ST36), Taichong（LR3）, according to the symptoms to add matching points, 2 Hz/100 Hz, density wave, 30min/d,6 times/week + neurology routine treatment | Sertraline hydrochloride tablet 20 mg/d+ routine treatment in neurology department |
| Peng 2009 | \ | China | RCT | Consistent with Chinese and Western medicine diagnosis of stroke, confirmed by CT and MRI; Meet the criteria of depression, no previous organic mental disease; Age 50 to 80 years old, agreed to be enrolled | 20≤HRSD score ≤35/SDS | Electroacupuncture three temporal acupuncture, Neiguan(PC6), Zusanli(ST36), Fenglong (ST40) and Taichong（LR3） (both sides were taken), density wave, 20min/ time, once/day, 6 times/week + fluoxetine hydrochloride 20mg/d+ routine treatment in neurology department | Fluoxetine hydrochloride 20mg/d+ routine treatment in neurology department |
| Peng 2011 | \ | China | RCT | Consistent with Chinese and Western medicine diagnosis of stroke, confirmed by CT and MRI; Meet the criteria of depression, no previous organic mental disease; Age 50 to 80 years old, agreed to be enrolled | 20≤HRSD score ≤35/SDS | Electroacupuncture of temporal three needles, Neiguan(PC6), Zusanli(ST36), Fenglong (ST40), Taichong（LR3） (both sides were taken), density wave, 20min/ time, 1 time/day, 6 times/week; Fluoxetine hydrochloride 20mg/d+ routine treatment in neurology department | Fluoxetine hydrochloride 20mg/d+ routine treatment in neurology department |
| Shao 2020 | 2016.8-2018.12 | China | RCT | Eligible for Chinese and Western stroke, 40 < age < 65, duration of disease < 3 months, agreed to be enrolled | Hamilton Depression Scale (HAMD) | Fluoxetine hydrochloride 20mg/d+ electroacupuncture at ear point heart, liver, kidney, brain stem and Shenmen(HT7), each time at one ear point, alternating point selection in both ears every other day, 15Hz, 30min/ time, once/day, 6 times/week | Fluoxetine hydrochloride 20mg/d |
| Sun 2018 | \ | China | RCT | Consistent with Chinese and Western stroke, confirmed by CT and MRI, more than half a month, within two years; Meet the criteria of depression, first after stroke, duration ≥2 weeks; 45≤ age ≤80; Clear awareness and agree to be included | 8 ≤HAMD score ≤35 | Electric acupuncture Sanyin Jiao(SP6) (bilateral), Yintang（GV24）, Shenmen(HT7) (bilateral), Sishen needle, Zhisan needle, density wave, 25min/ time, 1 time /d | Sertraline hydrochloride tablet 50mg/d |
| Tang2003 | 1998-2001 | China | RCT | CT confirmed cerebral stroke, not taking antidepressants | HAMD score ≥8 on 24 items | Electroacupuncture Baihui（GV20）, Shenting (DU24), Sishencong, Neiguan(PC6), Shenmen(HT7), Jiside take Neiguan(PC6), Shenmen(HT7), Zusanli(ST36), Taichong（LR3）, 1.3Hz-1.6Hz, 30min/ time,1 time/day, 20 times + routine treatment in neurology department | Rehabilitation treatment + conventional treatment in neurology department |
| Wa 2021 | 2017.10-2019.10 | China | RCT | In line with Chinese and Western medicine stroke, post-stroke depression; 40≤ age ≤85, agreed to be enrolled | 18 ≤HRSD score ≤35 | Electroacupuncture Baihui（GV20）, Sishencong, Ganshu(BL18), Sanyinjiao(SP6), Taichong（LR3）, 30min/ time, | Fake acupuncture without breaking through the skin |
| Wang 2013 | 2007.8-2009.8 | China | RCT | In line with traditional Chinese and Western medicine diagnosis of stroke, confirmed by CT and MRI, the first brain Within 2 months after stroke; Not taking antidepressants, and no other antidepressants and antipsychotics are used in combination with treatment; Clear awareness and consent to enrollment | HAMD score <17 | Efflafaxine 75mg/d; Electroacupuncture Du pulse meridian point, the main point to take Baihui（GV20）, Shenting (DU24), Fengfu (GV16), matching points to take Yintang（GV24）, three Yin jiao (SP6), 6V, Shubo, 2Hz, 30min/ times, 6 times a week, Hugh 1d | Venlafaxine 75mg/d |
| Wang 2015a | \ | China | RCT | CT and MRI confirmed cerebral infarction or cerebral hemorrhage patients, the duration of disease ≤3 months; Age ≤75, agreed to be enrolled | HAMD≥16 | Electroacupuncture Baihui（GV20）, Shenting (DU24), Neiguan(PC6), Shenmen(HT7), continuous wave, 2 Hz,30 min/ time, 1 time/day, + conventional treatment in neurology department | Sertraline hydrochloride 50 mg/d+ routine treatment in neurology |
| Wang 2015b | \ | China | RCT | CT and MRI confirmed cerebral infarction or cerebral hemorrhage patients, the course of disease ≤3 months; Age ≤76, agreed to be enrolled | HAMD≥16 | Electroacupuncture Baihui（GV20）, Shenting (DU24), Neiguan(PC6), Shenmen(HT7), continuous wave, 50 Hz,30 min/ time, 1 time/day, 30d+ routine treatment in neurology department | Sertraline hydrochloride 50 mg/d+ routine treatment in neurology |
| Wang 2015c | \ | China | RCT | The duration of cerebral infarction or cerebral hemorrhage confirmed by CT and MRI was ≤3 months; Age ≤77, agreed to be enrolled | HAMD≥16 | Electroacupuncture Baihui（GV20）, Shenting (DU24), Neiguan(PC6), Shenmen(HT7), continuous wave, 100Hz,30 min/ time, 1 time/day, 30d | Sertraline hydrochloride 50 mg/d+ routine treatment in neurology |
| Wang 2016 | 2008.3-2011.5 | China | RCT | Eligible for post-stroke depression of traditional Chinese and Western medicine, no delusional mental illness, no antidepressant treatment, agreed to be included in the group | HAMD Scale | Electroacupuncture ear point Shenmen(HT7), brain stem, heart, liver, kidney, left and right ear alternate points every other day, Baihui（GV20）, Shenmen(HT7), Sanyin Jiao(SP6), Taichong（LR3）, 30 min/ time, 1 time/day,5 times/week | Fluoxetine hydrochloride 20mg/d |
| Wang 2018 | 2015.3-2017.3 | China | RCT | The diagnosis of stroke was consistent with traditional Chinese and Western medicine. The course of the disease was confirmed by CT and MRI, ranging from 1 month to 6.5 years. Agreed to be enrolled | HAMD≥18 | Fluoxetine hydrochloride 20mg/d+ Xiaoyao SAN + electroacupuncture Fengchi(GB20) point, Jiaji point, current 2 mA, 30 min/time, 5 times/week, 10 times as a course of treatment, a total of 6 courses of treatment, each course interval of 2 days | Fluoxetine hydrochloride 20mg/d+ traditional Chinese medicine Xiaoyao SAN |
| Wang 2020 | 2016.4-2018.3 | China | RCT | It was confirmed by CT that cerebral infarction was in the convalescent stage, with symptoms of anxiety and depression, and agreed to be enrolled | 24 items Hamilton Depression Scale | Electroacupuncture Baihui（GV20）, Sishencong, 30min/ time, once /d, after 5 days, stop for 2 days + Chinese medicine Jieyu decoction + neurology routine treatment | Fluoxetine 20mg/d+ conventional treatment in neurology |
| Wang 2022 | \ | China | RCT | Consistent with Chinese and Western medicine diagnosis of stroke, confirmed by CT and MRI; Age: 35 ~ 80 years old, agreed to be enrolled | HAMD17 scores ≥16 | Electroacupuncture TouWei(GB13), ShenMen(HT7), 30min/ time, 5 times/week + psychological rehabilitation + basic treatment of neurology | Psychological rehabilitation + basic treatment in neurology |
| Wang 2023a | 2021.1-2023.1 | China | RCT | In line with Chinese and western medicine diagnosis of stroke, confirmed by CT and MRI; 40≤ age < 70, onset less than 1 year, more than 3 months and less than 1 year after stroke; Agreed to be enrolled | HAMD≥24 | Electroacupuncture Shenmen(HT7), Baihui（GV20）, Neiguan(PC6), Shanzhong(RN17), Yintang（GV24）, Taichong（LR3）, continuous wave, 2Hz,30min/ time, 1 time/day, 5 times/week + basic treatment of neurology | Non-current acupuncture Shenmen(HT7), Baihui（GV20）, Neiguan(PC6), Shanzhong(RN17), Yintang（GV24）, Taichong（LR3） + basic treatment of neurology |
| Wang 2023b | 2021.1-2023.1 | China | RCT | In line with Chinese and western medicine diagnosis of stroke, confirmed by CT and MRI; 40≤ age < 70, onset less than 1 year, more than 3 months and less than 1 year after stroke; Agreed to be enrolled | HAMD≥24 | Electroacupuncture Shenmen(HT7), Baihui（GV20）, Neiguan(PC6), Shanzhong(RN17), Yintang（GV24）, Taichong（LR3）, continuous wave, 2Hz,30min/ time, 1 time/day, 5 times/week + basic treatment of neurology | No current acupuncture Shenmen(HT7), Baihui（GV20）, Neiguan(PC6), Shanzhong(RN17), Yintang（GV24）, Taichong（LR3） |
| Wei 2010 | 2007.6-2010.6 | China | RCT | Consistent with Chinese and Western medicine diagnosis of stroke, confirmed by CT and MRI; No previous history of mental illness | HAMD Scale | Fluoxetine hydrochloride 20mg/d; Psychotherapy; Electroacupuncture Baihui（GV20）, Fengchi(GB20), Shenting (DU24), Neiguan(PC6), Shenmen(HT7), etc., density wave, 1Hz, 10-30Ma,20min/ time, 1 time/day, 10 times/course | Fluoxetine hydrochloride 20mg/d |
| Wen 2018 | 2015.6-2016.12 | China | RCT | In line with traditional Chinese and Western medicine diagnosis of stroke, confirmed by CT and MRI; 40≤ age ≤75; Did not take antidepressants and agreed to be enrolled | HAMD≥8 | Swallowing function rehabilitation training; Electroacupuncture Lianquan(CV23), Sishencong, Hegu（LI4）, Neiguan(PC6), Taichong（LR3）, Zusanli(ST36), Zhaohai(KI6), 20min/ time; NMES treatment; | Rehabilitation training |
| Xie 2017 | 2014.1-2016.12 | China | RCT | In line with traditional Chinese and Western medicine diagnosis of stroke, postoperative limb dysfunction accompanied by anxiety and depression symptoms 's patient | SDS | Electric needle Jianyu, Binao(LI14), Shou Sanli(ST36), Quchi(LI11), Hegu（LI4）, etc., Biguan(GB31), FuTu(BL40), Weizhong(BL40), Zusanli(ST36), JueGu, Xie Xi(KD8), Taichong（LR3）, etc., dredging wave, 30min/ time, 1 time /d+ basic rehabilitation therapy | Rehabilitation training |
| Xu 2014 | 2011.1-2013.6 | China | RCT | In line with traditional Chinese and Western medicine diagnosis of stroke, postoperative limb dysfunction accompanied by anxiety and depression symptoms 's patient | HAMD≥20 | Electroacupuncture Baihui（GV20）, Yintang（GV24）, Sishencong, Taichong（LR3）, Shenmen(HT7), Neiguan(PC6), Sanyinjiao(SP6), Taixi(KD3), Xinshu(BL15), continuous wave, frequency in 120 ~ 250 times /min, power 15 min, retention needle 30 min, 1 time /d, 14 Times for a course of treatment, a total of 3 courses of treatment. | Prozac capsules 20 mg/ day |
| Yang 2013a | \ | China | RCT | Consistent with Chinese and Western medicine diagnosis of stroke, confirmed by CT and MRI; No previous history of mental illness | HAMD | Electroacupuncture in frontal area, frontal area, Shan Zhong(RN17), Laogong(PC8) (double), Daling(PC7) (double), Anmian (double), Taichong（LR3） (double), Shumi wave, frequency 20-80Hz,30min/ time, 1 time /d, 6 times 1 course of treatment, rest 1d+ rehabilitation treatment + conventional treatment in neurology | Luyoutai 600mg/d, divided into two + rehabilitation treatment + conventional treatment |
| Yang 2013b | \ | China | RCT | Consistent with Chinese and Western medicine diagnosis of stroke, confirmed by CT and MRI; No previous history of mental illness | HAMD | Lu Youtai 600mg/d divided into two + electroacupuncture in frontal area, frontal area, Shan Zhong(RN17), Lao Gong (PC8)(double), Daling(PC7) (double), Anmian (double), Taichong（LR3） (double), dense wave, frequency 20-80Hz,30min/ times, 1 time /d, 6 times 1 course, rest 1d+ rehabilitation treatment + conventional treatment in neurology | Luyoutai 600mg/d, divided into two + rehabilitation treatment + conventional treatment |
| You 2013 | 2012.3-2013.3 | China | RCT | In line with traditional Chinese and Western medicine diagnosis of stroke, confirmed by CT and MRI; Consistent with depression, no previous history of mental illness; Age < 70 years, agreed to be enrolled | HAMD-17 score ≥17 | Electroacupuncture of bilateral Xin Shu(BL15), Ge Shu(BL17), Pi Shu(BL20), Gan Shu(BL18), Shen Shu(KD3), dredge wave, 30 min/ time, 1 time/day, 5 times/week, + paroxetine hydrochloride tablet 20 mg/d+ rehabilitation treatment + routine treatment in neurology | Paroxetine hydrochloride tablet 20 mg/d+ rehabilitation treatment + conventional neurological treatment |
| Yu 2005a | \ | China | RCT | Consistent with Chinese and Western medicine diagnosis of stroke, confirmed by CT and MRI; Consistent with depression, no previous history of mental illness, course of disease 2 weeks; Age 31-70, agreed to enroll | HAMD > 20, SDS > 41 | Electroacupuncture Neck Jiaji point, 6v, thin wave, 1Hz | Start at 20mg/d, no obvious discomfort after two weeks, 40mg/d, 28d a course of treatment, 4 months |
| Yu 2005b | \ | China | RCT | Consistent with Chinese and Western medicine diagnosis of stroke, confirmed by CT and MRI; Consistent with depression, no previous history of mental illness, course of disease 2 weeks; Age 31-70, agreed to enroll | HAMD > 20, SDS > 41 | Electroacupuncture Baihui（GV20）, Shenting (DU24), Sishencong, Yintang（GV24）, 6v, Shubo, 1Hz, 45min/ time | Start at 20mg/d, no obvious discomfort after two weeks, 40mg/d, 28d a course of treatment, 4 months |
| Yu 2013a | 2012.1-2012.12 | China | RCT | Meet the diagnostic standards of Chinese and Western medicine; HAMD≥7, ≤24; Cerebral hemorrhage or cerebral infarction confirmed by CT or MRI; The age of consciousness is 30-75 years old; The secondary stroke occurred 2 weeks after the acute stage, and the course of the disease was within half a year; Signed informed consent form | HAMD Scale | Electroacupuncture Baihui（GV20）, Shenting (DU24), Benshen(GV16), Fengchi(GB20), Shenmen(HT7), Neiguan(PC6), Hegu（LI4）, Taichong（LR3）, Zusanli(ST36), Sanyinjiao(SP6) and other acupoints, power, density wave, 2Hz, based on patient tolerance, 2 times/day, 6 days/week | Electroacupuncture without current |
| Yu 2013b | 2012.1-2012.12 | China | RCT | Meet the diagnostic standards of Chinese and Western medicine; HAMD≥7, ≤24; Cerebral hemorrhage or cerebral infarction confirmed by CT or MRI; The age of consciousness is 30-75 years old; The secondary stroke occurred 2 weeks after the acute stage, and the course of the disease was within half a year; Signed informed consent form | HAMD Scale | Music Curative hair + Electroacupuncture | Electroacupuncture without current |
| Zhang 2015a | \ | China | RCT | Eligible patients with post-stroke depression; Have not taken antidepressant treatment; Volunteer for a study. | HAMD Scale/SDS | Oral Chinese medicine 150ml, 2 times/day; Electroacupuncture Shenting (DU24), Baihui（GV20）, Sishencong, Neiguan(PC6), Yanglingquan(GB34), Zusanli(ST36), Sanyinjiao(SP6), etc., power, 6V, density wave, 2HZ, for patient tolerance, once/day, 30min/ times | Oral Chinese medicine |
| Zhang 2015b | \ | China | RCT | Eligible patients with post-stroke depression; Have not taken antidepressant treatment; Volunteer for a study. | HAMD Scale/SDS | Oral Chinese medicine 150ml, 2 times/day; Electroacupuncture Shenting (DU24), Baihui（GV20）, Sishencong, Neiguan(PC6), Yanglingquan(GB34), Zusanli(ST36), Sanyinjiao(SP6), etc., power, 6V, density wave, 2HZ, for patient tolerance, once/day, 30min/ times | Oral fluoxetine, 20mg/ time, once/day |
| Zhang 2016 | 2012.11-2015.8 | China | RCT | Met the diagnostic criteria of stroke depression; HAMD≥18 | HAMD Scale | Oral citalopram tablet 20mg+ electroacupuncture Baihui（GV20）, Yintang（GV24）, electrification, once a day, 45mIn/ times, 5 times/week | Oral citalopram tablet 20mg, once /d, 1 tablet/time up to 40mg/d |
| Zhang 2017 | 2016.5-2017.2 | China | RCT | Meet the diagnostic criteria for depressive episode; HAMD > 16; Age < 75; No history of mental illness; Signed informed consent form | HAMD Scale | Neurology routine treatment plus electroacupuncture Shanzhong(RN17), Hegu（LI4）, Neiguan(PC6), Taixi(KD3), Xuan Zhong, Sanyinjiao(SP6), electricity, tolerance strength for patients; Leave for 30min, once a day | Routine treatment in neurology department was supplemented with oral fluoxetine hydrochloride 20mg once per day |
| Zhou 2007 | \ | China | RCT | Stable and conscious; Meet the diagnostic criteria of post-stroke depression; HAMD ≥ 20; And no history of mental illness | HAMD Scale | Electric acupuncture Baihui（GV20）, Yintang（GV24）, power, voltage 6v, 2HZ, once a day, 45Min/ times | Oral fluoxetine hydrochloride, starting at 10mg/d, starting at 20mg/d on the fourth day |
| Zhou 2011 | 2010.9-2010.12 | China | RCT | Stroke occurred for the first time with CT or MRI meeting the diagnostic criteria; SCL-90≥160; The course of the disease is more than 2 weeks conscious; Hemilimb motor dysfunction; Informed consent | Self-rating SDS Scale for depression | Routine treatment of neurology plus electric acupuncture Jianyu(LI15), Quchi(LI11), Shou Sanli(ST36), Waiguan(SJ5), Hegu（LI4）,Bi Guan(GB31),HuanTiao(GB30),Fu Tu(BL40), Zusanli(ST36), Yanglingquan(GB34), electrifying 20min, density wave, 1 time. Day, 10 times/course, plus music therapy 30min | Routine treatment in neurology plus supportive psychological care |
| Zuo 2023 | 2020.42021.12 | China | RCT | In line with the diagnostic criteria of Chinese and Western medicine, the first onset was post-stroke depression; Age: 30-75 years old, duration ≤6 months; Voluntary informed consent form | HAMD Scale/SDS | Electroacupuncture Tianquan (LU9), Quze(LI11), Neiguan(PC6), Daling(PC7), electrifying for 30Min, based on patient tolerance, 2/20hz density wave; 1 time/day, 5 times/week + neurological treatment | Acupuncture without current + neurological treatment |

Table S3 Research characteristics (continue)

| **study** | **patients** | | **Duration of intervention** | **male** | | **duration of the disease** | | **Mean/median Age（years)** | | **Outcoms** |
| --- | --- | --- | --- | --- | --- | --- | --- | --- | --- | --- |
|  | **Intervention group** | **Control group** |  | **Intervention group** | **Control group** | **Intervention group** | **Control group** | **Intervention group** | **Control group** |  |
| Bi 2009a | 31 | 32 | 6 Weeks | \ | \ | \ | \ | \ | \ | HAMD、SDS |
| Bi 2009b | 31 | 32 | 6 Weeks | \ | \ | \ | \ | \ | \ | HAMD、SDS |
| Bi 2009c | 32 | 32 | 6 Weeks | \ | \ | \ | \ | \ | \ | HAMD、SDS |
| Bi 2009d | 31 | 32 | 6 Weeks | \ | \ | \ | \ | \ | \ | HAMD、SDS |
| Cai 2019 | 29 | 29 | 4 weeks | 18 | 16 | \ | \ | 67.62±11.14 | 67.66±11.56 | adverse reaction rate、Overall efficacy rate、HAMD、TMC-DS |
| Cai 2020a | 30 | 30 | 4 weeks | 17 | 18 | 5.54±1.34 months | 5.52±1.21months | 62.25±3.57 | 61.88±3.82 | Overall efficacy rate、HAMD |
| Cai 2020b | 30 | 30 | 4 weeks | 17 | 19 | 5.54±1.34 months | 5.46±1.26 months | 62.25±3.57 | 62.43±3.28 | Overall efficacy rate、HAMD |
| Chang 2011 | 50 | 50 | 4 weeks | 30 | 29 | \ | \ | \ | \ | Overall efficacy rate、HAMD |
| Chen 2012 | 47 | 47 | 12 weeks | \ | \ | \ | \ | \ | \ | HAMD |
| Chen 2014 | 30 | 30 | 9 weeks | 14 | 17 | 151.07±9.98days | 150.08±8.82days | 53.9±8.09 | 54.1±7.89 | HAMD、SDS |
| Cheng 2007Ⅰa | 20 | 19 | 6 Weeks | 9 | 8 | \ | \ | 63.2±7.9 | 61.7±8.1 | adverse reaction rate、HAMD、SDS |
| Cheng 2007Ⅰb | 20 | 19 | 6 Weeks | 9 | 10 | \ | \ | 63.2±7.9 | 62.9±7.3 | adverse reaction rate、HAMD、SDS |
| Cheng 2007Ⅱ a | 20 | 19 | 6 Weeks | 9 | 8 | \ | \ | 69±7 | 72±8 | adverse reaction rate、HAMD |
| Cheng 2007Ⅱ b | 20 | 21 | 6 Weeks | 9 | 10 | \ | \ | 69±7 | 69±6 | adverse reaction rate、HAMD |
| Cui 2008 | 30 | 30 | 6 Weeks | 19 | 20 | \ | \ | 65.47±10.32 | 68.23±10.30 | Overall efficacy rate、HAMD、TCM-DS |
| Deng 2016 | 49 | 45 | 6 Weeks | \ | \ | \ | \ | \ | \ | Overall efficacy rate、HAMD |
| Ding 2020 | 61 | 61 | 4 weeks | 30 | 29 | 5.22±1.07months | 5.16±1.02months | 54.63±3.51 | 54.27±3.43 | Overall efficacy rate、HAMD |
| Ding 2023a | 40 | 40 | 6 Weeks | 27 | 26 | 6.23±3.25months | 6.23±3.23months | 63.35±7.04 | 63.25±7.12 | adverse reaction rate、、Overall efficacy rate、HAMD |
| Ding 2023b | 40 | 40 | 6 Weeks | 27 | 28 | 6.23±3.25months | 6.28±3.25months | 63.35±7.04 | 63.28±7.16 | adverse reaction rate、Overall efficacy rate、HAMD |
| Dong 2007Ⅰ | 38 | 38 | 4 weeks | 21 | 20 | \ | \ | 62.4 | 61.6 | adverse reaction rate、Overall efficacy rate、HAMD |
| Dong 2007Ⅱa | 38 | 36 | 4 weeks | 25 | 23 | 2.60±2.20 months | 2.80±2.01 months | 58.40±9.60 | 59.21±7.56 | Overall efficacy rate、HAMD、SDS |
| Dong 2007Ⅱb | 38 | 34 | 4 weeks | 25 | 19 | 2.60±2.20 months | 2.50±2.10 months | 58.40±9.60 | 56.61±8.21 | Overall efficacy rate、HAMD、SDS |
| Dong 2016a | 15 | 15 | 6 Weeks | \ | \ | \ | \ | \ | \ | HAMD |
| Dong 2016b | 15 | 15 | 6 Weeks | \ | \ | \ | \ | \ | \ | HAMD |
| Dong 2017a | 50 | 50 | 4 weeks | \ | \ | \ | \ | \ | \ | Overall efficacy rate、HAMD、SDS |
| Dong 2017b | 50 | 50 | 4 weeks | \ | \ | \ | \ | \ | \ | Overall efficacy rate、HAMD、SDS |
| Feng 2012 | 45 | 45 | 6 Weeks | 27 | 25 | 27.4 days | 25.8 days | 54.2 | 52.7 | HAMD、SDS |
| Gao 2013 | 30 | 30 | 8 weeks | 16 | 18 | 3.21±1.98 months | 3.27±1.87 months | 59.56±10.45 | 56.25±10.38 | adverse reaction rate、Overall efficacy rate、HAMD |
| Gao 2013‖ | 30 | 30 | 4 weeks | 16 | 14 | \ | \ | 58.69±8.34 | 59.11±3.25 | HAMD |
| Guo 2011a | 32 | 31 | 6 Weeks | \ | \ | \ | \ | \ | \ | HAMD |
| Guo 2011b | 32 | 31 | 6 Weeks | \ | \ | \ | \ | \ | \ | HAMD |
| He 2020 | 23 | 23 | 4 weeks | 14 | 13 | \ | \ | 62.24±9.72 | 60.23±10.45 | Overall efficacy rate、HAMD |
| Hong 2015 | 30 | 30 | 30 days | \ | \ | \ | \ | \ | \ | adverse reaction rate、Overall efficacy rate、HAMD |
| Huang 2005 | 30 | 30 | 6 Weeks | 17 | 16 | 1.14±0.42 months | 1.33±0.28 months | 61.43±9.91 | 62.10±8.11 | adverse reaction rate、Overall efficacy rate、HAMD |
| Huang 2014a | 30 | 30 | 6 Weeks | 17 | 16 | 1.14±0.42 months | 1.16±0.30 months | 61.43±9.91 | 62.77±9.32 | adverse reaction rate、Overall efficacy rate、HAMD |
| Huang 2014b | 46 | 44 | 6 Weeks | 17 | 16 | 1.14±0.42 months | 1.16±0.30 months | 61.43±9.91 | 62.77±9.32 | adverse reaction rate、Overall efficacy rate、HAMD |
| Huang 2019 | 100 | 100 | 4 weeks | 53 | 55 | 3.41±0.62 months | 3.23±0.58 months | 6011±2.25 | 60.32±2.18 | HAMD、SDS |
| Jiang 2006 | 37 | 38 | 4 weeks | 21 | 20 | \ | \ | 62.5±12.4 | 61.8±13.5 | adverse reaction rate、HAMD |
| Jiang 2017 | 31 | 30 | 4 weeks | 17 | 18 | 3.78±2.46 months | 3.62±2.54 months | 60.32±3.26 | 61.18±2.94 | Overall efficacy rate、HAMD |
| Jiao 2018 | 49 | 48 | 6 Weeks | 30 | 26 | \ | \ | 60.8±7.2 | 61.3±7.2 | HAMD |
| Kang 2014 | 40 | 40 | 4 weeks | 26 | 29 | \ | \ | \ | \ | HAMD |
| Li 2013 | 35 | 35 | 4 weeks | 19 | 22 | \ | \ | \ | \ | Overall efficacy rate、HAMD |
| Liu 2015 Ⅰ | 30 | 30 | 4 weeks | 20 | 18 | 10.5±2.5 days | 11.2±1.9 days | 61. 5 ± 5. 6 | 50.3 ± 5.5 | Overall efficacy rate、HAMD |
| Liu 2015 Ⅱ | 30 | 30 | 8 weeks | 20 | 18 | 10.5±2.5 days | 11.2±1.9 days | 61.5±5.6 | 50.3±5.5 | Overall efficacy rate、HAMD、SDS |
| Liu 2016 | 43 | 43 | 4 weeks | 26 | 26 | \ | \ | 62.4±7.2 | 62.4±7.2 | HAMD |
| Liu 2021 | 30 | 30 | 8weeks | 17 | 18 | \ | \ | 58±9 | 58±8 | HAMD |
| Long 2004 | 36 | 36 | 4 weeks | 24 | 23 | \ | \ | 59.8±7.6 | 60．1±6．9 | HAMD |
| Meng 2023 | 39 | 39 | 4 weeks | 25 | 26 | 25.49 ± 2.91 days | 26.18±2.01 days | 67.00 ±5.99 | 66.31 ±5.73 | Overall efficacy rate、HAMD |
| Ni 2023 | 32 | 34 | 8 weeks | 17 | 20 | 6.4±2.0 months | 6.4±1.8 months | 68±9 | 68±9 | adverse reaction rate、Overall efficacy rate、HAMD |
| Peng 2009 | 30 | 30 | 4 weeks | 21 | 20 | \ | \ | 64.5±11.2 | 63.9±11.3 | HAMD、SDS |
| Peng 2011 | 58 | 59 | 4 weeks | 38 | 40 | \ | \ | 64.6±11.3 | 73.7±11.8 | adverse reaction rate、HAMD、SDS |
| Shao 2020 | 40 | 40 | 6 Weeks | 23 | 25 | 19.34 ± 4.37days | 18.28 ± 5.36days | 54.36 ± 6.31 | 54.64 ± 8.21 | Overall efficacy rate、HAMD |
| Sun 2018 | 33 | 33 | 4 weeks | 19 | 17 | 11.88±4.76 months | 13.39±4.15 months | 57.94±7.55 | 58.42±7.42 | HAMD、TCM-DS |
| Tang2003 | 30 | 30 | 4 weeks | 19 | 20 | 19±15 months | 19±15 months | 66.97±7.75 | 66.03±7.42 | HAMD |
| Wa 2021 | 33 | 32 | 4 weeks | 20 | 18 | 2.0±1.26 years | 2.3±1.49 years | 67.8±10.91 | 66.7±11.42 | HAMD、SDS、TCM-DS |
| Wang 2013 | 35 | 38 | 8 weeks | 17 | 21 | 23.21±10.26 days | 22.51±9.56 days | 48.21±9.34 | 47.53±8.92 | HAMD |
| Wang 2015a | 25 | 25 | 4 weeks | 12 | 15 | 72.25±2.53 days | 71.87±2.12 days | 68±2 | 72±2 | Overall efficacy rate、HAMD |
| Wang 2015b | 25 | 25 | 4 weeks | 13 | 15 | 73.65±2.76 days | 71.87±2.12 days | 67±2 | 72±2 | Overall efficacy rate、HAMD |
| Wang 2015c | 25 | 25 | 4 weeks | 11 | 15 | 72.73±2.06 days | 71.87±2.12 days | 68±2 | 72±2 | Overall efficacy rate、HAMD |
| Wang 2016 | 49 | 49 | 8 weeks | 22 | 25 | 2.1±0.3 months | 1.9±0.5 months | 62±6 | 60±5 | Overall efficacy rate、HAMD |
| Wang 2018 | 75 | 75 | 6weeks |  |  | 1.8 ±0.33 years | 1.3±0.27 years | 56.3 ± 3.11 | 57.2 ± 3.25 | HAMD |
| Wang 2020 | 43 | 43 | 8 weeks | 28 | 25 | 3.27 ± 2.31 months | 3.13 ± 2.18 months | 51.16 ± 10.34 | 50.81 ± 10.85 3 | adverse reaction rate、Overall efficacy rate、HAMD |
| Wang 2022 | 26 | 23 | 12 weeks | 14 | 12 | 10.9 ± 1.1 months | 15.5 ± 1.5 months | 60.3 ± 5.3 | 59.8 ± 5.5 6 | HAMD |
| Wang 2023a | 25 | 25 | 4 weeks | 18 | 17 | 6.833±2.20 months | 6.667±2.20 months | 54.167±8.042 | 56.208±7.108 | Overall efficacy rate、HAMD |
| Wang 2023b | 25 | 25 | 4 weeks | 15 | 17 | 7.120±2.333 months | 6.667±2.200 months | 54.080±7.686 | 56.208±7.108 | Overall efficacy rate、HAMD |
| Wei 2010 | 42 | 42 | 4 weeks | 23 | 22 | 4.6±2.5 months | 5.1±2.3 months | 57.2±10.8 | 56.1±11.4 | HAMD |
| Wen 2018 | 46 | 45 | 4 weeks | 26 | 24 | 22.38±11.63 days | 21.66±12.35 days | 55.62±14.34 | 54.65±13.87 | HAMD |
| Xie 2017 | 40 | 40 | 6 Weeks | 23 | 24 | 2.8±0.7 months | 2.3±0.6 months | 58.1±6.0 | 58.7±6.5 | SDS |
| Xu 2014 | 40 | 40 | 6 Weeks | 18 | 21 | \ | \ | 66±10 | 65±9.8 | Overall efficacy rate、HAMD |
| Yang 2013a | 20 | 20 | 56d | 9 | 9 | \ | \ | \ | \ | Overall efficacy rate、HAMD |
| Yang 2013b | 20 | 20 | 56d | 9 | 8 | \ | \ | \ | \ | Overall efficacy rate、HAMD |
| You 2013 | 30 | 30 | 4 weeks | 20 | 18 | 3weeks-2months | 3weeks-2months | 42.38 | 43.17 | Overall efficacy rate、HAMD |
| Yu 2005a | 30 | 29 | 4weeks | 17 | 15 | \ | \ | 61.7±8.13 | 62.85±7.32 | HAMD、SDS |
| Yu 2005b | 28 | 29 | 4weeks | 16 | 15 | \ |  | 63.1±7.91 | 62.85±7.32 | HAMD、SDS |
| Yu 2013a | 30 | 30 | 4 weeks | 16 | 18 | 2.45±0.72 months | 2.48±0.91 months | 64.60±7.24 | 64.07±8.45 | adverse reaction rate、Overall efficacy rate、HAMD |
| Yu 2013b | 30 | 30 | 4 weeks | 16 | 17 | 2.45±0.72 months | 2.57±0.78 months | 64.60±7.24 | 65.10±6.20 | adverse reaction rate、Overall efficacy rate、HAMD |
| Zhang 2015a | 20 | 20 | 8 weeks | \ | \ | \ | \ | \ | \ | HAMD、SDS |
| Zhang 2015b | 20 | 20 | 8 weeks | \ | \ | \ | \ | \ | \ | HAMD、SDS |
| Zhang 2016 | 30 | 30 | 6 Weeks | 18 | 19 | 1.02±0.73 years | 1.06±0.59 years | 57.69±6.90 | 56.81±7.36 | Overall efficacy rate、HAMD |
| Zhang 2017 | 35 | 35 | 4 weeks | \ | \ | \ | \ | \ | \ | Overall efficacy rate、HAMD |
| Zhou 2007 | 31 | 30 | 4 weeks | 20 | 22 | 13.42±2.86 months | 13.42±2.86 months | 64.82±5.63 | 64.12±9.63 | HAMD |
| Zhou 2011 | 36 | 32 | 4weeks | 28 | 26 | 9.5±3.1 months | 8.9±2.6 months | 56.6±12.3 | 55.8±11.7 | SDS |
| Zuo 2023 | 25 | 25 | 8 weeks | 14 | 16 | 83.24±52.893 days | 86.2±64.626 days | 61.24±9.320 | 56.00±11.383 | Overall efficacy rate、HAMD、SDS |
