# Supplementary material for: Efficacy and safety of electroacupuncture-based comprehensive treatment for post-stroke depression: a systematic review and meta-analysis of randomized controlled trials
Source: Front Psychiatry. 2025 Aug 15;16:1610032. doi: 10.3389/fpsyt.2025.1610032 (PMC12395381; doi:10.3389/fpsyt.2025.1610032)
Supplement: Supplementary file 4 [file Table4.doc]

Table S4 Acupoint frequency

| Acupoint name | Serial number | English name | Meridians | Main functions | Treatment | Frequency |
| --- | --- | --- | --- | --- | --- | --- |
| 百会 | GV20 | Baihui | Conception Vessel | The main functions of regulating emotions, invigorating qi and rising Yang, and promoting orifices and relieving pain | Headache, vertigo, insomnia, mental tension | 41 |
| 神庭 | GV24 | Shenting | Conception Vessel | regulating emotions, clearing heat and detoxifying, improving memory and sleep | Headache, insomnia, memory loss, lack of concentration, epilepsy, syncope, neurasthenia, anxiety, depression | 28 |
| 太冲 | LR3 | Tai Chong | Liver Meridian of Foot-Jueyin, LR | Soothing the liver and regulating qi, clearing the liver and brightening the eyes, regulating qi and blood, calming and regulating emotions | Headache, vertigo, eye fatigue, blurred vision, chest tightness, flank distention pain, irregular menstruation, dysmenorrhea, hypertension, insomnia | 28 |
| 神门 | HT7 | Shenmen | Heart Meridian of Hand-shaoyin, HT | regulating emotions Regulating the nervous system | Insomnia, anxiety, heart disease | 26 |
| 内关 | PC6 | Neiguan | Heart Meridian of Hand-shaoyin, HT | It regulates Pericardium Meridian of Hand-Jueyin, PC, dreads meridians, calms regulating emotions, and regulates gastrointestinal function | Chest pain, palpitation, coronary heart disease, insomnia, anxiety, epilepsy, nausea, vomiting, stomachache, stomach distension, relieving vomiting, often used for nausea after chemotherapy | 22 |
| 印堂 | GV24 | Yintang (GV24) | Conception Vessel | Calming and regulating emotions, clearing heat and detoxification, and improving blood circulation in the brain | Relieve anxiety, insomnia, nervous system related diseases, relieve dizziness, lack of blood supply to the brain, remove the heat of the head and face, relieve headache, eye discomfort | 22 |
| 合谷 | LI4 | Hegu | Yangming Large Intestine Channel of Hand | Dissipate wind, relieve pain, regulate visceral function and enhance immunity | Treat cold, headache, fever, toothache, headache, neck stiffness, dyspepsia | 17 |
| 三阴交 | SP6 | Sanyinjiao | Spleen Meridian of Foot-Taiyin, SP | Regulating spleen and stomach, soothing liver and relieving depression, tonifying kidney and fixing essence, regulating menstruation and relieving pain | Irregular menstruation, constipation, diarrhea, frequent urination, insomnia | 14 |
| 足三里 | ST36 | Zusanli | Stomach Meridian of Foot-Yangming, ST | Nourishing qi and blood, regulating spleen and stomach, improving immunity and relieving fatigue | Treatment of appetite loss, dyspepsia, abdominal pain and abdominal distension; Chronic gastritis, constipation, chronic fatigue | 14 |
| 本神 | GV16 | Benshen | Gallbladder Meridian of Foot-Shaoyang, GB | Soothing the liver and Qianyang, relieving wind and spasmolysis, dredging collaterals and relieving pain | ‌ Epilepsy, headache, dizziness, insomnia, chest and hypochondriac pain | 8 |
| 风池 | GB20 | Fengchi | Gallbladder Meridian of Foot-Shaoyang, GB | Dissipating wind, clearing heat and detoxifying, activating blood circulation, regulating emotions and determination | Treat cold, headache and strong neck pain caused by exogenous wind and cold, wind and heat; Treatment of throat pain, tinnitus and dizziness caused by wind and heat; Arthralgia, migraine, cervical spondylosis; Anxiety, insomnia | 7 |
| 率谷 | SJ5 | Shuai Gu | Gallbladder Meridian of Foot-Shaoyang, GB | Soothe the liver and relieve depression and regulating emotions | Mental illness, emotional instability | 6 |
| 强间 | ST3 | Qiang Jian | Conception Vessel | Improves cerebral blood circulation, regulating emotions, and relieves pain | Headache, mental strain, insomnia | 6 |
| 曲池 | LI11 | Quchi | Yangming Large Intestine Channel of Hand | Clearing heat and detoxifying, promoting blood circulation and removing blood stasis, and regulating immunity | High fever, skin diseases, arthralgia, elbow pain, stroke sequelae, constipation | 6 |
| 阳白 | GB14 | Yangbai | Gallbladder Meridian of Foot-Shaoyang, GB | Clearing heat and detoxifying, dispelling wind and dispersing cold, harmonizing qi and blood, dredging meridians, smoothing the head and face, improving vision and eye health | Headache, migraine, red eye swelling and pain, eye diseases (such as eye fatigue, dizziness), facial numbness, facial nerve paralysis, cold and cold, influenza, nasal congestion, rhinitis, toothache, gum swelling and pain | 6 |
| 脑户 | GV15 | Brain's Door | Conception Vessel | Awaken your mind, improve your eyesight, and improve your blood circulation | Headache, dizziness, dizziness, insomnia, confusion, epilepsy, memory loss, anxiety, hypertension caused by headache | 5 |
| 曲鬓 | GB6 | Shuaigu | Gallbladder Meridian of Foot-Shaoyang, GB | Refresh the mind, clear the liver and clear the eyes | Relieve headache, vertigo, tinnitus, insomnia, mental restlessness, facial neuralgia, trigeminal neuralgia | 5 |
| 头临泣 | GB1 | Toulinqi | Gallbladder Meridian of Foot-Shaoyang, GB | Clearing heat and detoxifying, relieving wind and pain, and improving vision | Treatment of eye diseases (such as redness, swelling and pain, blurred vision, etc.), headache, facial neuralgia, migraine, high intraocular pressure, reduce vertigo | 5 |
| 悬厘 | GB6 | Xuanli | Gallbladder Meridian of Foot-Shaoyang, GB | Dissipating wind, clearing heat and detoxifying, promoting blood circulation and removing blood stasis | Headache, dizziness, tinnitus, neck stiffness, shoulder and back pain, wind and heat cold, acute tonsillitis, cervical spondylosis, blood stasis after sports injury | 5 |
| 悬颅 | GB5 | Xuanlu | Gallbladder Meridian of Foot-Shaoyang, GB | Clearing heat and detoxifying, regulating emotional sedation, and regulating brain function | Headache, insomnia, vertigo, neurasthenia, eye disease | 5 |
| 髀关 | GB 31 | Biguan | Stomach Meridian of Foot-Yangming, ST | Dispelling wind and relieving pain, promoting blood circulation and removing blood stasis, and relieving pain | Lower limb numbness, pain, joint inflexibility, rheumatic diseases, lumbago and leg pain | 4 |
| 伏兔 | BL40 | Futu | Stomach Meridian of Foot-Yangming, ST | Promoting blood circulation, relieving pain, detoxifying and detumescence | Treatment of lower limb spasticity, leg soreness, knee joint pain, heel pain; Treatment of hemorrhoids, constipation, sciatica; Relieve poor blood circulation in lower limbs | 4 |
| 肩髃 | LI15 | Jianyu | Yangming Large Intestine Channel of Hand | Jianyu Shujin activates collaterals, regulates the qi and blood of the Yangming Large Intestine Channel of Hand, and enhances the local circulation of qi and blood | Shoulder soreness, joint stiffness, shoulder periarthritis | 4 |
| 手三里 | ST36 | Hand San Li | Stomach Meridian of Foot-Yangming, ST | Tonifying qi and nourishing blood, regulating spleen and stomach, enhancing physical strength, promoting digestion and absorption, relieving gastrointestinal discomfort, enhancing immunity, and resisting fatigue | Stomach pain, stomach distension, vomiting, diarrhea, constipation, frequent cold, lack of physical strength, high blood lipids, high blood sugar, relieve anxiety, insomnia, fatigue | 4 |
| 肝俞 | BL18 | Ganshu | Bladder Meridian of Foot-Taiyang | Soothe the liver and relieve depression, clear the liver fire, and the liver and gallbladder meridians | Treatment of liver qi stagnation, liver fire rise, liver stagnation and spleen deficiency caused by various symptoms; Treatment of hepatitis, cholecystitis, dyspepsia, headache, dizziness, liver depression and qi stagnation | 3 |
| 解溪 | KD8 | Jiex | Stomach Meridian of Foot-Yangming, ST | Clearing heat and reducing fire, relaxing tendon and activating collaterality, and reducing swelling with water | Headache, vertigo, red eyes, abdominal distension, constipation, madness, head and face swelling, lower limb paralysis, ankle weakness | 3 |
| 膻中 | RN17 | Shan Zhong | Conception Vessel | Shan Zhong regulates qi machine, regulates qi by broadening chest, soothe emotions and promotes qi and blood circulation | Treat chest tightness and shortness of breath, palpitations, cough and wheezing, breast problems, and emotional problems such as anxiety and depression | 3 |
| 外关 | SJ5 | Wai Guan | Sanjiao Meridian of Hand-shaoyang, SJ | Dissipate wind, relieve pain and clear heat | Cold, cold, shoulder pain, headache | 3 |
| 阳陵泉 | GB34 | Yanglingquan | Gallbladder Meridian of Foot-Shaoyang, GB | Soothing the liver and promoting the gallbladder, activating meridians and relieving pain | Headache, vertigo, flank pain, treatment of leg, hip pain, numbness and movement disorders, treatment of muscle, joint and spinal diseases, treatment of cholecystitis, dyspepsia, jaundice | 3 |
| 大陵 | PC7 | Daling | Pericardium Meridian of Hand-Jueyin, PC | Analgesic and soothing | Palpitations, anxiety, and insomnia | 2 |
| 环跳 | GB30 | Huantiao | Bladder Meridian of Foot-Taiyang | Relieves pain and improves circulation | Relieve lower limb pain, spasticity, numbness, low back pain, hip pain, sciatica, regulate gynecological diseases and improve blood circulation | 2 |
| 梁丘 | ST34 | Liang Qiu | Stomach Meridian of Foot-Yangming, ST | Invigorate qi and blood circulation, detoxify and detumescence | Abdominal pain, indigestion, constipation | 2 |
| 头维 | GB13 | Tou Wei | Stomach Meridian of Foot-Yangming, ST | Clearing heat and detoxifying, activating collaterals and relieving pain, and balancing qi and blood | Headache, vertigo, migraine, eye disease, eye pain, eye fatigue, facial paralysis, treatment of emotional disorders, insomnia | 2 |
| 心俞 | BL15 | Xin Shu | Bladder Meridian of Foot-Taiyang | regulating heart function, dredging heart pulse, regulating emotions | Treatment of palpitations, insomnia, chest pain, shortness of breath | 2 |
| 阴陵泉 | SP9 | Yinlingquan | Spleen Meridian of Foot-Taiyin, SP | Clearing damp qi, regulating spleen and stomach, regulating digestive system, regulating qi and relieving pain, nourishing Yin liquid | Edema, loss of appetite, dyspepsia, abdominal distension, abdominal pain, constipation and diarrhea, urgency, frequent urination, and painful urination | 2 |
| 大椎 | GV14 | Dazhui | Conception Vessel | Shujin activates collaterals, activates blood and relieves pain, and promotes qi-blood circulation | Lower limb numbness, muscle spasm, sciatica, lower limb edema | 2 |
| 臂臑 | LI14 | Binao | Yangming Large Intestine Channel of Hand | Binao activates blood circulation to remove blood stasis and relieve pain by dredging collaterals | Relieve shoulder pain and stiffness, treat shoulder periarthritis and shoulder movement disorder | 1 |
| 胆俞 | BL19 | Dan Shu | Bladder Meridian of Foot-Taiyang | Soothing liver and gallbladder, regulating spleen and stomach, clearing heat and dampness, dredging meridians | Cholecystitis, cholelithiasis, flank pain, jaundice, weakness of spleen and stomach, dyspepsia, vomiting, chest and back pain | 1 |
| 丰隆 | ST40 | Fenglong | Stomach Meridian of Foot-Yangming, ST | Fenglong regulates the spleen and stomach, Shujin and activates collaterals, dissipates phlegm and stops coughing | Treatment of spleen and stomach discord, phlegm dampness, dyspepsia, treatment of cardiovascular and cerebrovascular diseases, hyperlipidemia, hypertension | 1 |
| 风府 | GV16 | Fengfu | Conception Vessel | Dissipating wind, clearing heat and phlegm, regulating emotions, calming and waking up | Treatment of rheumatism, cold pathogen invasion, cerebrovascular diseases; Stroke, epilepsy, vertigo; Insomnia, anxiety; Confusion and coma | 1 |
| 膈俞 | BL17 | Ge Shu | Bladder Meridian of Foot-Taiyang | It promotes the circulation of qi and blood, regulates the spleen and stomach, regulates qi and widens the chest, and activates blood and channels veins | Stomach flatulence, constipation, vomiting, hiccup, choking, chest fullness, flank pain, stomach pain, madness, hemoptysis, hematemesis, anemia, back pain | 1 |
| 公孙 | SP4 | Gongsun | Spleen Meridian of Foot-Taiyin, SP | Regulating the spleen and stomach, dredging qi and blood, relieving pain and safe fetus | Weakness of spleen and stomach, loss of appetite, abdominal distension; Abdominal pain and flank pain; Discomfort during pregnancy, such as fetal restlessness and abdominal pain | 1 |
| 劳宫 | PC8 | Laogong | Pericardium Meridian of Hand-Jueyin, PC | Laogong cleans and regulates emotions, detoxifies and detumescence, improves circulation and regulates body temperature | Treatment of insomnia, anxiety, irritability caused by strong heart fire, treatment of chest tightness, palpitations, mouth and tongue sores, auxiliary treatment of heat stroke, fever, treatment of fever, headache | 1 |
| 廉泉 | CV23 | Lienquan | Conception Vessel | Dredge qi and blood, clear heat and detoxify, relieve swelling and pain | Treatment of sore throat, thyroid disease, cough, cervical lymph node enlargement | 1 |
| 脾俞 | BL20 | Spleen Shu | Bladder Meridian of Foot-Taiyang | Regulate the spleen and stomach, nourish the spleen, strengthen the spleen and dissipate food | Loss of appetite, dyspepsia, abdominal distension, diarrhea, edema, edema, chronic fatigue, qi deficiency, obesity, back pain | 1 |
| 期门 | LV14 | Qimen | Liver Meridian of Foot-Jueyin, LR | Soothing liver and relieving depression, regulating qi machine, and stomach digestion, promoting blood circulation | Chest and hypochondrium pain, depression, chest tightness, epigastric pain, loss of appetite, nausea, dysmenorrhea, irregular menstrual period, headache, vertigo, chest pain | 1 |
| 曲泽 | LI5 | Qu Ze | Pericardium Meridian of Hand-Jueyin, PC | Dispel heat, open collaterals, and promote blood flow | Back pain, knee pain, low back pain, lower limb numbness, weakness, acute lumbar sprain, eczema, acne, urinary tract infection, kidney disease | 1 |
| 申脉 | DU14 | Shenmai | Bladder Meridian of Foot-Taiyang | Improves lung function and boosts immunity | Asthma, wheezing, shortness of breath | 1 |
| 肾俞 | KD3 | Shen Shu | Bladder Meridian of Foot-Taiyang | Tonifying the kidney, strengthening the essence, nourishing qi and blood, strengthening the waist, regulating the lower focus, adjusting the function of the kidney, and promoting the recovery of kidney qi | Low back pain, fatigue, dizziness, insomnia, spermatorrhea, impotence, irregular menstruation, chronic kidney disease, frequent urination, urgency, tinnitus, hearing loss, sexual dysfunction | 1 |
| 水沟 | ST9 | Shuigou | Conception Vessel | Shuigou regulates qi and blood, restores zangfu functions, relieves heat symptoms, clears heat and detoxicates, and refreshed the mind | Acute coma, syncope, shock, stroke sequela, epilepsy, convulsion, fever, high fever, acute poisoning | 1 |
| 太溪 | KD3 | Taixi | Kidney Meridian of Foot-shaoyin, KI | Nourishing kidney Yin, strengthening kidney qi, moistening lung, nourishing essence, regulating emotions | Waist and knee soreness, tinnitus and deafness, insomnia, spermatorrhea, cough | 1 |
| 天冲 | GB16 | Tian Chong | Gallbladder Meridian of Foot-Shaoyang, GB | Dissipating wind and clearing heat, clearing the ears and eyes, activating collaterals and channeling meridians, regulating qi and blood, and relieving pain | Headache, dizziness, tinnitus, deafness, facial neuralgia, neck pain, shoulder stiffness, insomnia, anxiety | 1 |
| 天泉 | LU9 | Taiyuan | Pericardium Meridian of Hand-Jueyin, PC | Clearing heat and detoxifying, moistening lung and relieving cough, activating qi and blood, regulating lung qi | Cough, expectoration, sore throat, acute and chronic bronchitis, bronchial asthma, lung heat cough, hoarse voice, lung deficiency, shortness of breath | 1 |
| 委中 | BL40 | Weizhong | Bladder Meridian of Foot-Taiyang | Weizhong clears heat and detoxicates, activates blood circulation and removes blood stasis, Shujin and activates collaterals | Treatment of low back pain, knee pain, cystitis, urinary retention, lower limb numbness, etc | 1 |
| 行间 | LR2 | Xingjian | Liver Meridian of Foot-Jueyin, LR | Soothing the liver and regulating qi, clearing the liver fire, regulating menstruation and regulating emotions | Irregular menstruation, dysmenorrhea, headache, vertigo, insomnia, anxiety, eye discomfort | 1 |
| 翳风 | GB 4 | Yi Feng | Sanjiao Meridian of Hand-shaoyang, SJ | Dissipating wind, clearing heat and lowering blood pressure | Headache, tinnitus, deafness, facial paralysis | 1 |
| 照海 | KI 6 | Zhaohai | Kidney Meridian of Foot-shaoyin, KI | Nourishing Yin and tonifying kidney, clearing heat and reducing fire, nourishing smart eyes, regulating emotions, improving edema, and regulating water metabolism | Treatment of eye diseases, waist and knee soreness, falls and injuries | 1 |
