# Supplementary material for: Efficacy and safety of electroacupuncture-based comprehensive treatment for post-stroke depression: a systematic review and meta-analysis of randomized controlled trials
Source: Front Psychiatry. 2025 Aug 15;16:1610032. doi: 10.3389/fpsyt.2025.1610032 (PMC12395381; doi:10.3389/fpsyt.2025.1610032)
Supplement: Supplementary file 5 [file Table5.doc]

Table S5 Sensitivity analysis

| STAT DATA | | | HAMD |
| --- | --- | --- | --- |
|  | | | 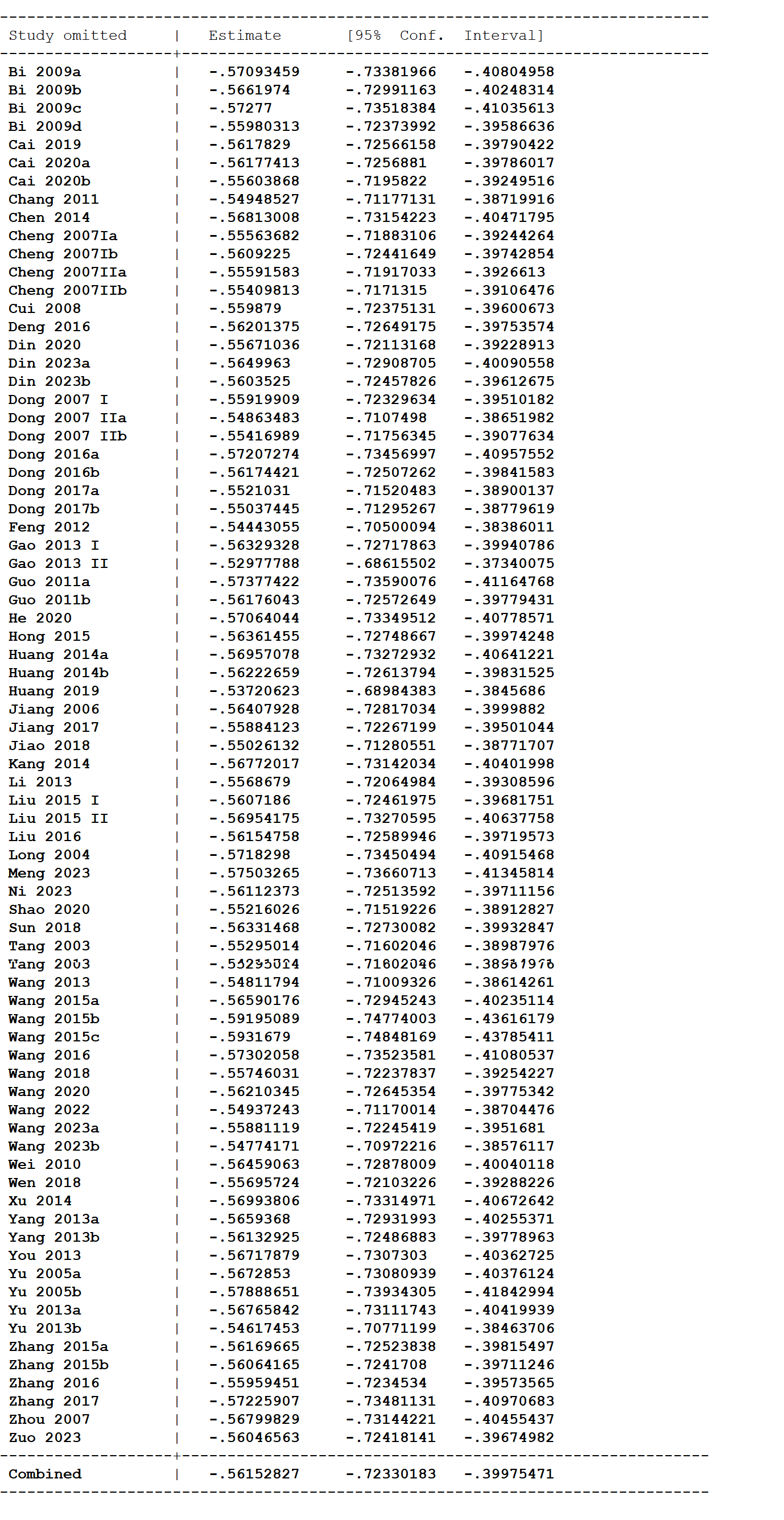 |
| 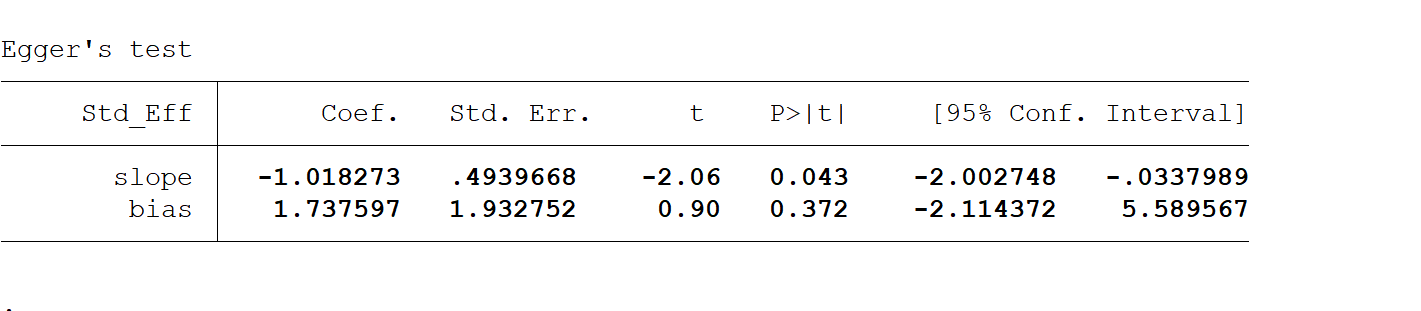 | | | |
| 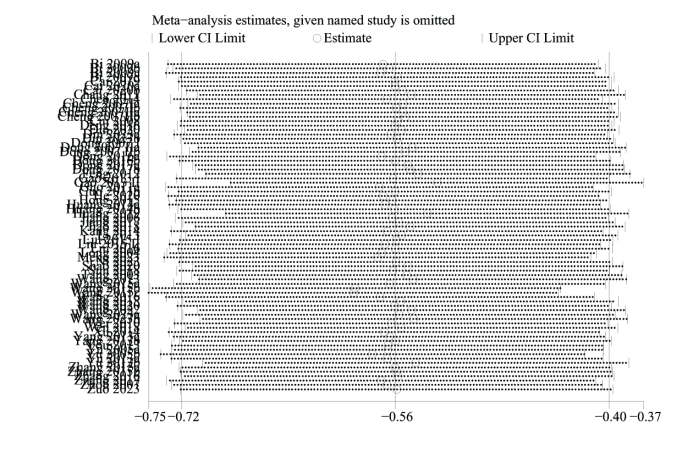 | | | |
| STAT DATA | SDS | | |
| 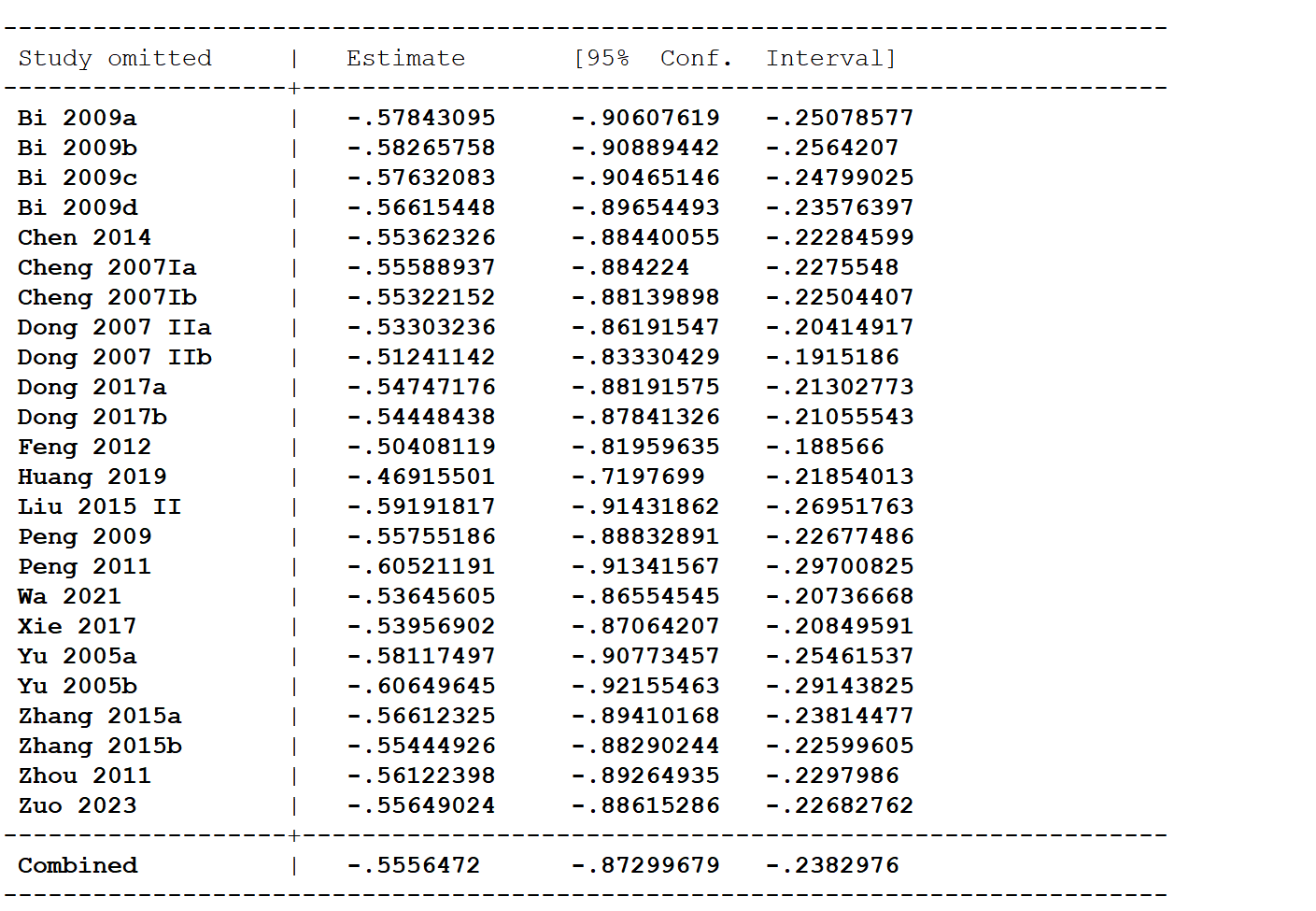 | | | |
| 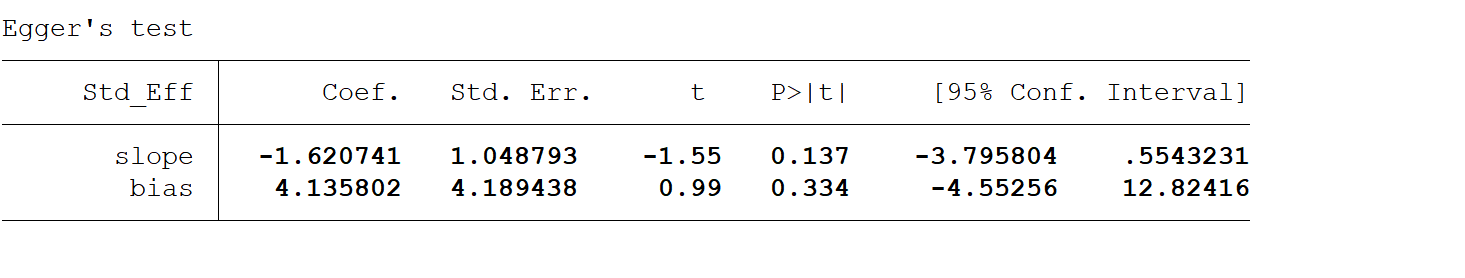 | | | |
| 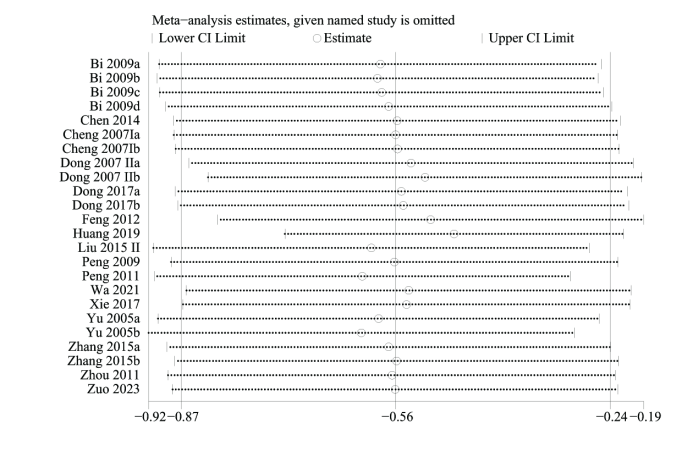 | | | |
| STAT DATA | | TCM-DS | |
| 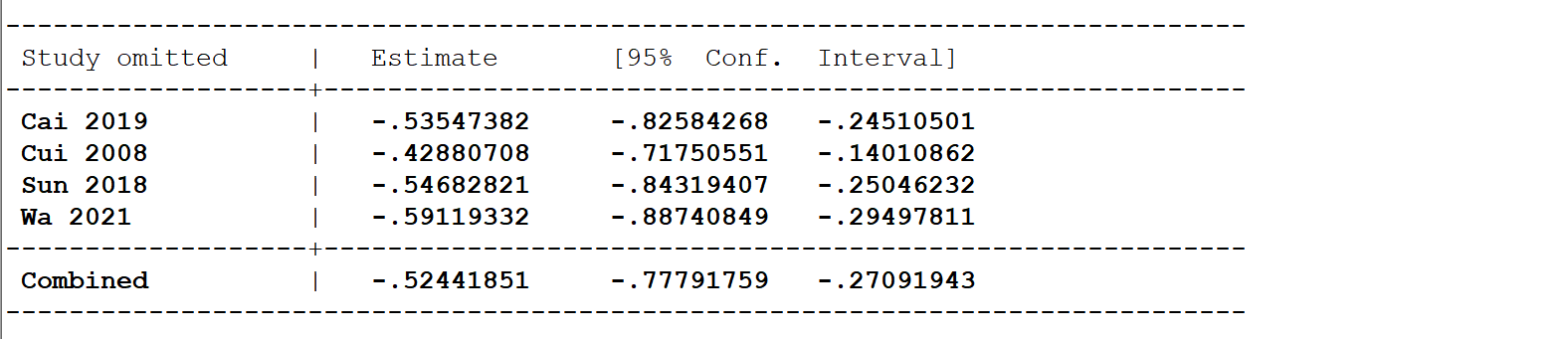 | | | |
| 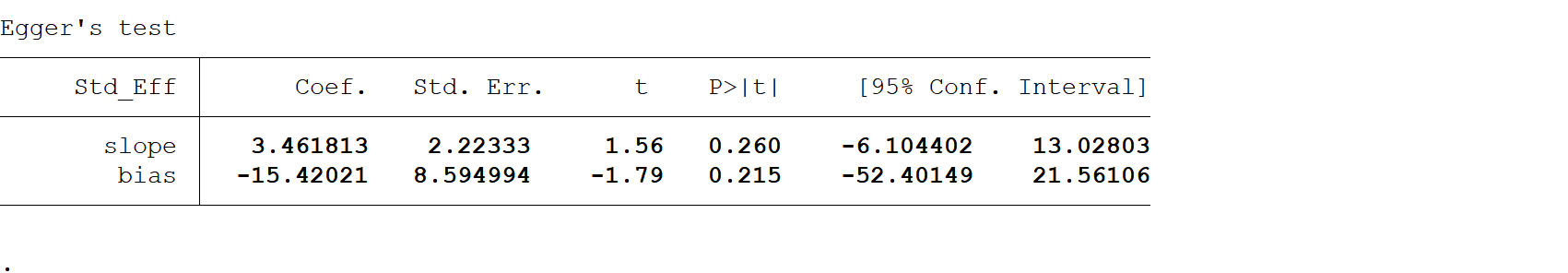 | | | |
| 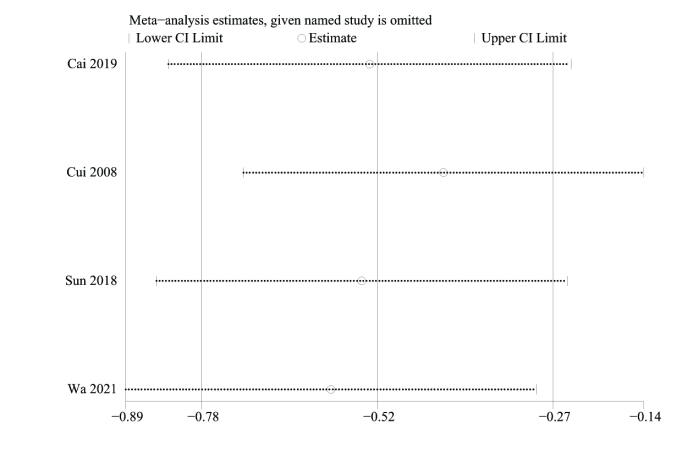 | | | |
| STAT DATA | | overall efficacy rate | |

| 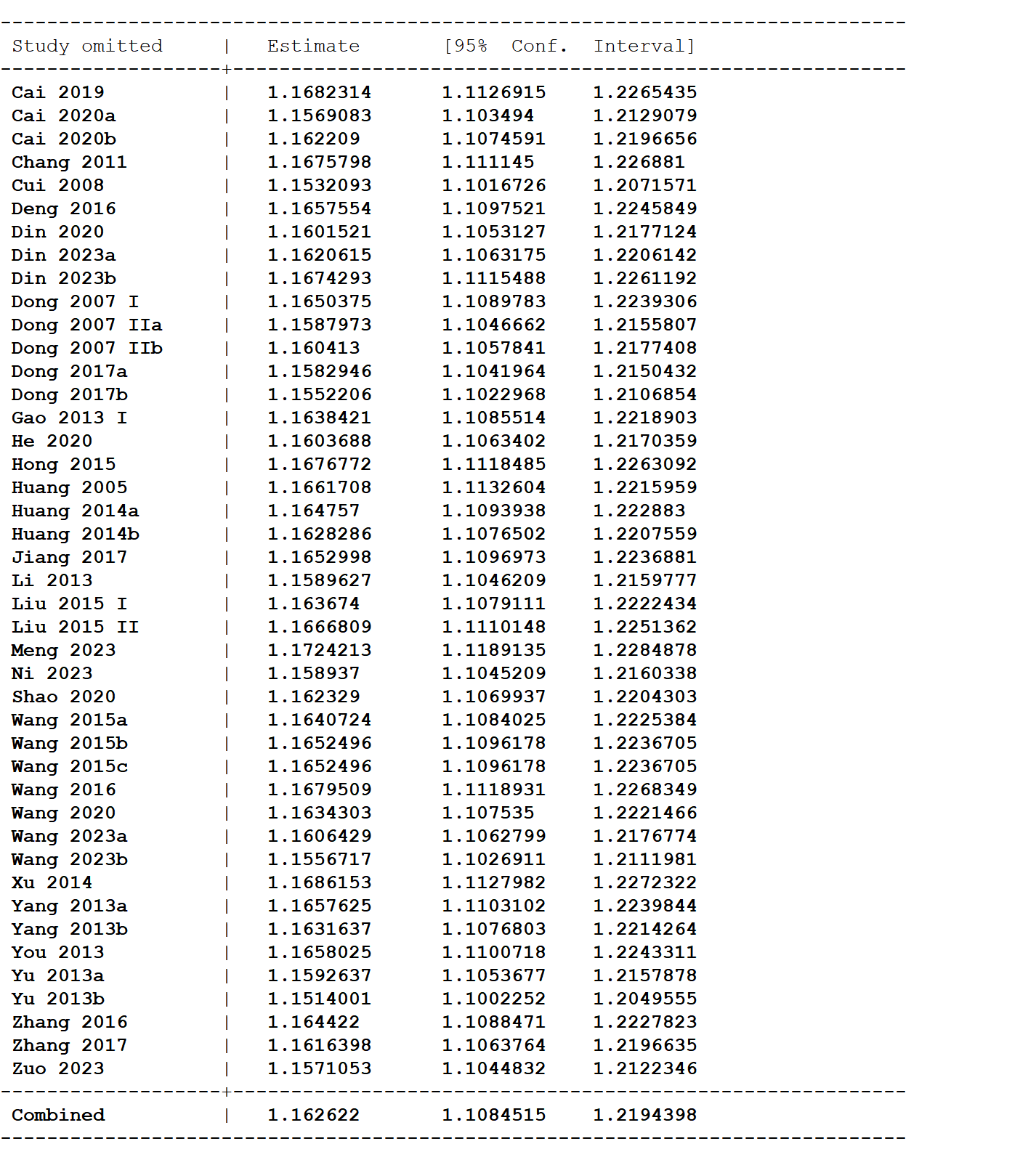 |
| --- |
| 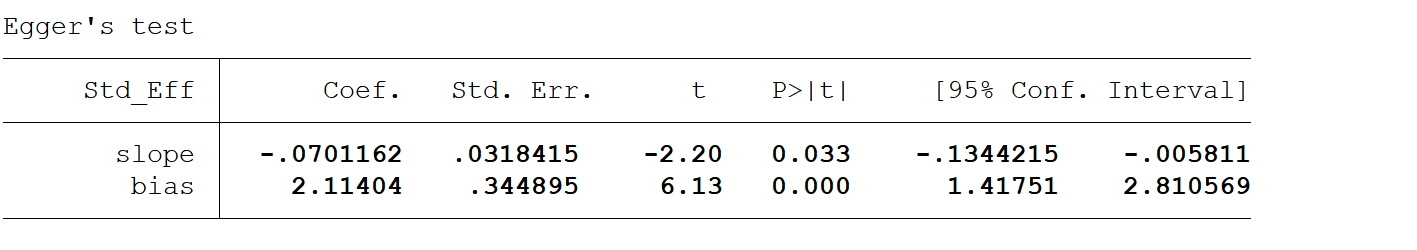 |
| 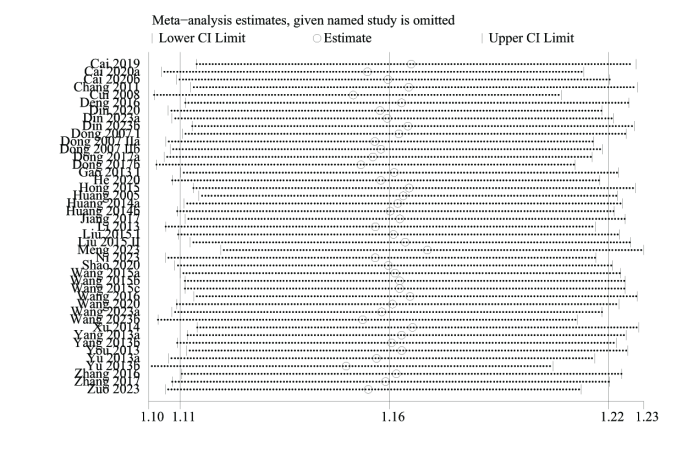 |

| STAT DATA  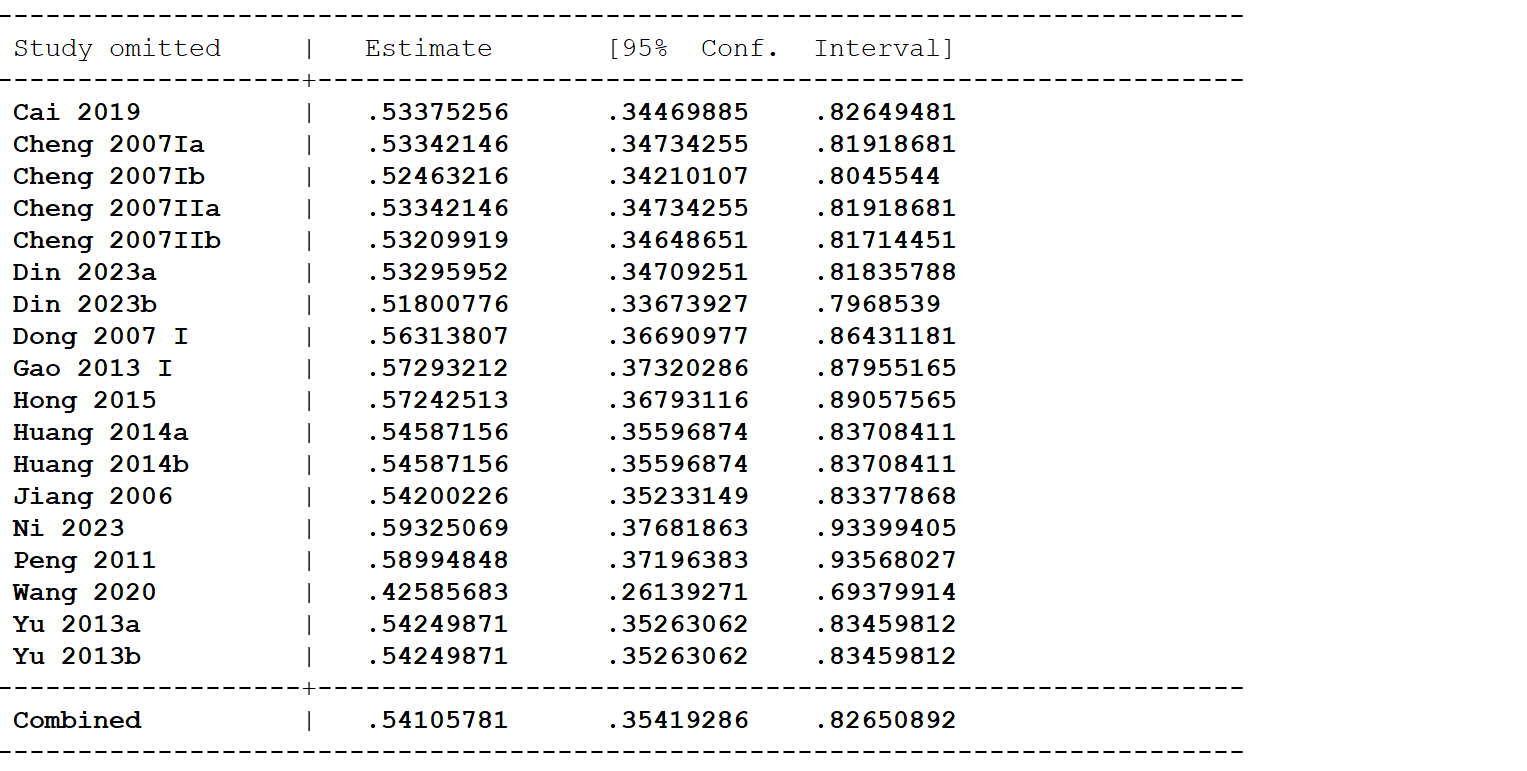   | adverse reaction rate |  | | --- | --- | |
| --- | --- | --- |
| 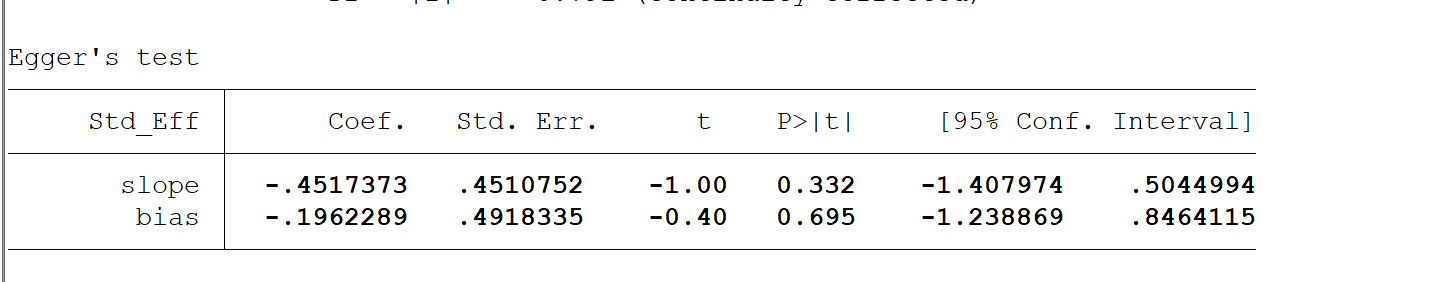 |
| 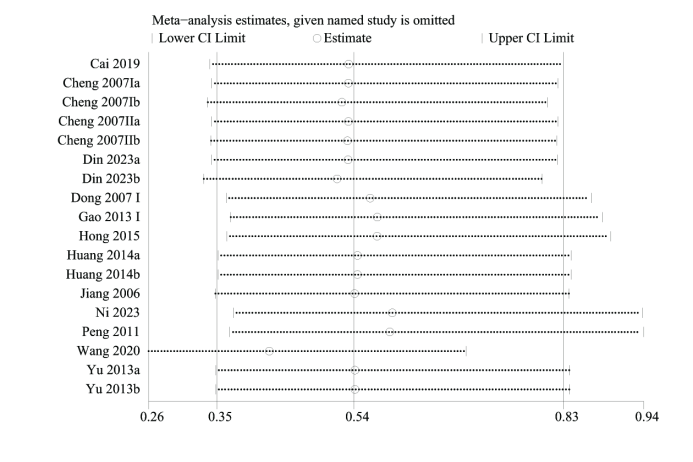 |
